# AMP-activated protein kinase promotes breast cancer stemness and drug resistance

**DOI:** 10.1242/dmm.049203

**Published:** 2022-05-27

**Authors:** Sai Balaji Andugulapati, Ananthalakshmy Sundararaman, Mohini Lahiry, Annapoorni Rangarajan

**Affiliations:** Department of Molecular Reproduction, Development and Genetics; Indian Institute of Science, Bangalore 560012, India

**Keywords:** AMPK, Anchorage deprivation, Stemness, Drug resistance, Twist

## Abstract

Breast cancer stem cells (BCSCs) are a major cause of therapy resistance and tumour progression. Currently, their regulation is not entirely understood. Previous work from our laboratory demonstrated a context-specific pro-tumorigenic role for AMP-activated protein kinase (AMPK) under anchorage-deprivation and mammosphere formation, which are hallmarks of BCSCs. Therefore, we investigated the role of AMPK in the maintenance of BCSC state/function. AMPK depletion reduces serial sphere formation *in vitro* and tumour initiation *in vivo*. Intriguingly, tumour-derived cell analysis using stem cell markers and functional assays revealed that AMPK is required for the maintenance of BCSC populations *in vivo*. AMPK promotes the expression of stemness genes such as *NANOG*, *SOX2* and *BMI1* through the transcriptional upregulation of *TWIST* via promoter acetylation. Further, AMPK-driven stemness plays a critical role in doxorubicin resistance. Significantly, AMPK activity increased after chemotherapy in patient-derived tumour samples alongside an increase in stemness markers. Importantly, AMPK depletion sensitises mouse tumours to doxorubicin treatment. Our work indicates that targeting of AMPK in conjunction with regular chemotherapy is likely to reduce the stem cell pool and improve chemosensitivity in breast cancers.

## Abstract (precis)

**Summary:** AMPK inhibition in conjunction with regular chemotherapy is likely to reduce the stem cell pool and improve chemosensitivity and therapeutic outcomes in breast cancers.

## INTRODUCTION

The success of anti-cancer therapies is often limited by the development of drug resistance. Resistance to drugs is facilitated by intratumoural heterogeneity, and the concept of cancer stem cells (CSCs) provides a framework for understanding one of the dimensions of this intratumoural heterogeneity (Meacham and Morrison, 2013). Phenotypically distinct cancer cells in the tumour are organized in a hierarchy that is very similar to the stem cell hierarchy of the corresponding non-neoplastic tissue. The CSCs at the apex of this hierarchy can give rise to the complex neoplastic tissue under appropriate conditions. Being slow dividing, these CSCs are not targeted by regular chemotherapy and, therefore, are responsible for further relapse and tumour progression, as well as metastasis. Several lines of evidence suggest the wide applicability of the CSC concept in different cancers, and this additionally guides therapy (Kreso and Dick, 2014). Several signalling pathways critical to stem and progenitor cell homeostasis and function, such as the Notch, Wnt, Hedgehog and Hippo signalling pathways, have been targeted to eliminate CSC-driven cancer progression (Malta et al., 2018). This study explores the role of AMP-activated protein kinase (AMPK; encoded by *PRKAA2*) in regulating BCSCs.

AMPK is a metabolic enzyme that is well known for its role in energy homeostasis whereby it switches off anabolic processes while turning on catabolism during bioenergetic stress conditions (Hardie, 2011). It is also centrally placed in the LKB1-AMPK-mTOR pathway (Shackelford and Shaw, 2009), which negatively regulates cell proliferation (Shaw, 2009). Together, these findings have largely associated AMPK with tumour suppressive functions. In contrast, we and others have recently shown elevated AMPK activity in high-grade breast cancer patients (Hart et al., 2015; Sundararaman et al., 2016). Moreover, AMPK is activated under diverse micro-environmental stress conditions encountered by cancer cells, such as glucose deprivation, hypoxia and matrix-deprivation, and brings about cancer cell survival, thus aiding tumour progression (Hindupur et al., 2014; Jeon et al., 2012; Kumar and Rangarajan, 2009; Saha et al., 2018). More recently, genetic studies of AMPK in murine cancer models have revealed that before the disease arises, AMPK acts as a tumour suppressor that protects against tumour initiation. Once the cancer has arisen, AMPK can switch to being a tumour promoter by enhancing cancer cell survival under various upstream metabolic, genotoxic or oxidative stresses (Vara-Ciruelos et al., 2019).

Matrix detachment leads to cell death by anoikis, and anoikis resistance is fundamental for cancer metastasis (Kim et al., 2012). Supporting the emerging role of AMPK as a contextual tumour promoter, we demonstrated that AMPK is required for the metabolic adaptation to matrix deprivation (Saha et al., 2018; Sundararaman et al., 2020). Anoikis resistance of cancer cells is achieved by AMPK activation-mediated PEA15 phosphorylation (Hindupur et al., 2014) and NADPH homeostasis by inhibition of acetyl-CoA carboxylase (ACC) (Jeon et al., 2012), as well as the direct inhibition of mammalian target of rapamycin complex 1 (mTORC1) (Ng et al., 2012). In our present study, we explore another facet of AMPK-dependent tumour promotion in the context of anchorage deprivation, namely an increase in BCSC pool.

The role of AMPK in cancer stemness is tissue-type specific and context dependent. Several discrepancies exist in the literature regarding whether it promotes or hinders cancer stemness. Breast cancer stem cells (BCSCs) are reported to utilize the AMPK-HIF1α pathway during state transitions to switch to a high oxidative phosphorylation state (Luo et al., 2018). Other studies have shown that AMPK negatively regulates CSCs in different cancer types through the inhibition of protein prenylation, reactive oxygen species-dependent autophagy and direct Nanog degradation (Seo et al., 2020; Wu et al., 2019; Zhao et al., 2019). In contrast, some studies have correlated AMPK activation with stemness through both the modulation of bioenergetics (Chhipa et al. 2018) and an increase of stemness factors, e.g. the nuclear PKM2 (also known as PKM) translocation and Oct4 (also known as POU5F1) induction (Yang et al., 2018).

Several of the studies outlined above use small molecule activators, such as metformin, to come to conclusions about the role of AMPK in cancer promotion or suppression. Small-molecule activators lead to supraphysiological AMPK activation, as well as having AMPK-independent effects (Ben Sahra et al., 2011). Yet others have used glucose deprivation as a stimulus to trigger AMPK, but multiple metabolic and signalling amplification loops are triggered by glucose deprivation (Graham et al., 2012), several of which are likely to be independent of AMPK. To overcome these issues, we chose to exploit anchorage deprivation as a cancer-specific and *in vivo*-like pathophysiologically relevant upstream activating signal for the AMPK pathway. We hypothesized that AMPK-dependent metabolic rewiring would favour a switch to an increased stemness phenotype in breast cancer cells under specific micro-environmental cues that activate it, such as anchorage deprivation.

Doxorubicin/adriamycin is a widely used adjuvant and neoadjuvant chemotherapeutic drug for breast cancer. Cells that have undergone an epithelial-mesenchymal transition (EMT) display multidrug resistance in a Twist (also known as TWIST1)-dependent manner (Li et al., 2009). Our work has previously demonstrated that AMPK can stabilize Twist protein, thereby promoting EMT (Saxena et al., 2018). In breast cancer, the EMT state is often associated with CSC properties, such as the expression of CD44^high^/CD24^low^ antigenic profile, self-renewal capabilities and resistance to conventional therapies (May et al., 2011). This led us to further hypothesize that the AMPK-Twist axis could govern CSC fate in breast cancers.

In this study, we provide evidence linking the AMPK-Twist axis to breast cancer stemness and adaptive drug resistance. Furthermore, we provide a direct mechanistic link between AMPK activation and *TWIST* gene expression through the acetylation of its promoter. Indeed the AMPK-Twist axis promotes the expression of ABC transporters implicated in multidrug resistance in breast cancer. Our study suggests that AMPK inhibition can improve the therapeutic outcome of regular chemotherapy by preventing the adaptive transition of cancer cells to a stem cell phenotype.

## RESULTS

### AMPK enriches for BCSCs *in vitro* and increases tumour initiation potential *in vivo*

**Fig. 1.**
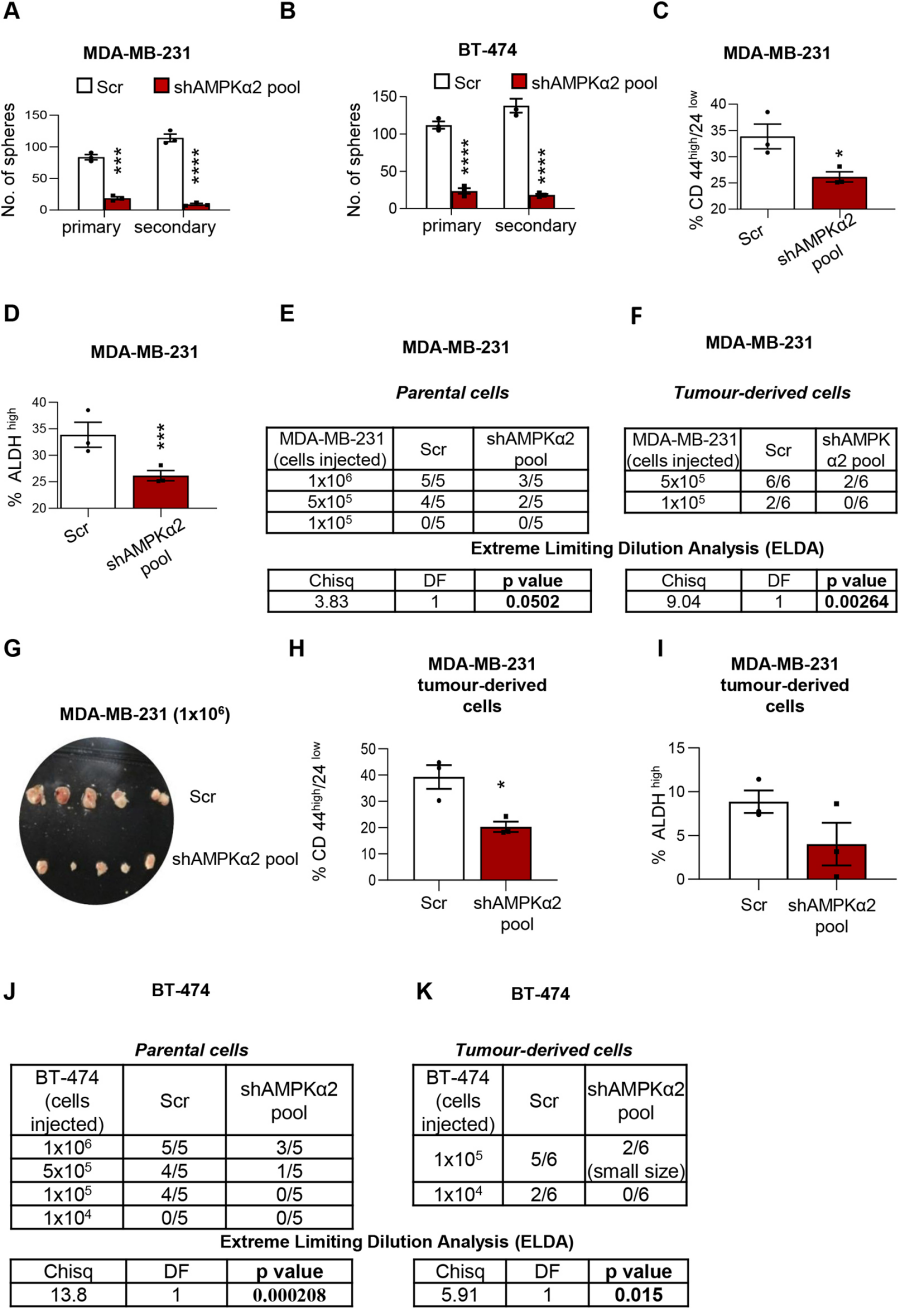
**AMPK enriches for BCSCs *in vitro* and increases tumour initiation potential *in vivo*.** (A,B) MDA-MB-231 (A) or BT-474 (B) cells stably expressing shRNA against AMPKα2-pool or scrambled shRNA (Scr) were seeded in methyl cellulose for sphere formation. After 1 week, the number of spheres (primary) were counted, trypsinized and reseeded (single cells) in methyl cellulose and incubated for another week for secondary sphere formation. (C,D) MDA-MB-231 cells stably expressing shRNA against AMPKα2-pool or scrambled shRNA (Scr) were cultured in suspension for 72 h, harvested and subjected to CD44^high^/24^low^ (C) and ALDH assay analysis (D). *n*=3. Representative FACS plots are featured in the Supplementary Materials and Methods. (E,F) MDA-MB-231 cells stably expressing shRNA against AMPKα2-pool or scrambled shRNA (Scr) were injected (10^5^, 5×10^5^ and 10^6^) subcutaneously into five female nude mice (control cells in the left flank, and shAMPKα2 cells in the right flank for each dilution), and tumour formation was assessed at 42 days, after which ELDA was performed. (F) Cells derived from both scr and shAMPKα2 tumours (isolated from 10^6^ injected set of mice) were re-injected in an independent set of nude mice, and tumour formation was assessed at 42 days for ELDA analysis. (G-I) MDA-MB-231 cells stably expressing shRNA against AMPKα2-pool or scrambled shRNA (Scr) were injected (10^5^, 5×10^5^ and 10^6^) subcutaneously into five female nude mice (control cells in the left flank, and shAMPKα2 cells in the right flank for each dilution) and assessed for tumour formation. After a specified time period, tumours were isolated and imaged (G), and tumour cells (10^6^ cells injected set of mice) were derived from the tumours and subjected to CD44^high^/24^low^ analysis (H) and ALDH assay analysis (I). (J,K) BT-474 cells stably expressing shRNA against AMPKα2-pool or scrambled shRNA (Scr) were injected (10^4^, 10^5^_,_ 5×10^5^, 10^6^) subcutaneously into five female nude mice (for each cell dilution). Scr cells were injected in the in the left flank, and shAMPKα2-pool cells in the right flank. Tumour formation was assessed at 35 days post injection. The number of palpable tumours (minimum 100mm^3^ in size) in each case are represented in the table and ELDA was performed. For *in vivo* passaging, tumour-derived cells (10^6^ cells injected set of mice) were collected and re-injected into a new set of female nude mice, and tumour formation was assessed at day 35, after which ELDA analysis was performed. Data are mean±s.e.m (*n*=3). *P<0.05, ***P<0.001, ****P<0.001 (two-way ANOVA, unpaired two-tailed Student's *t*-test or χ^2^ (chisq) tests were performed for statistical significance).

**Fig. 2.**
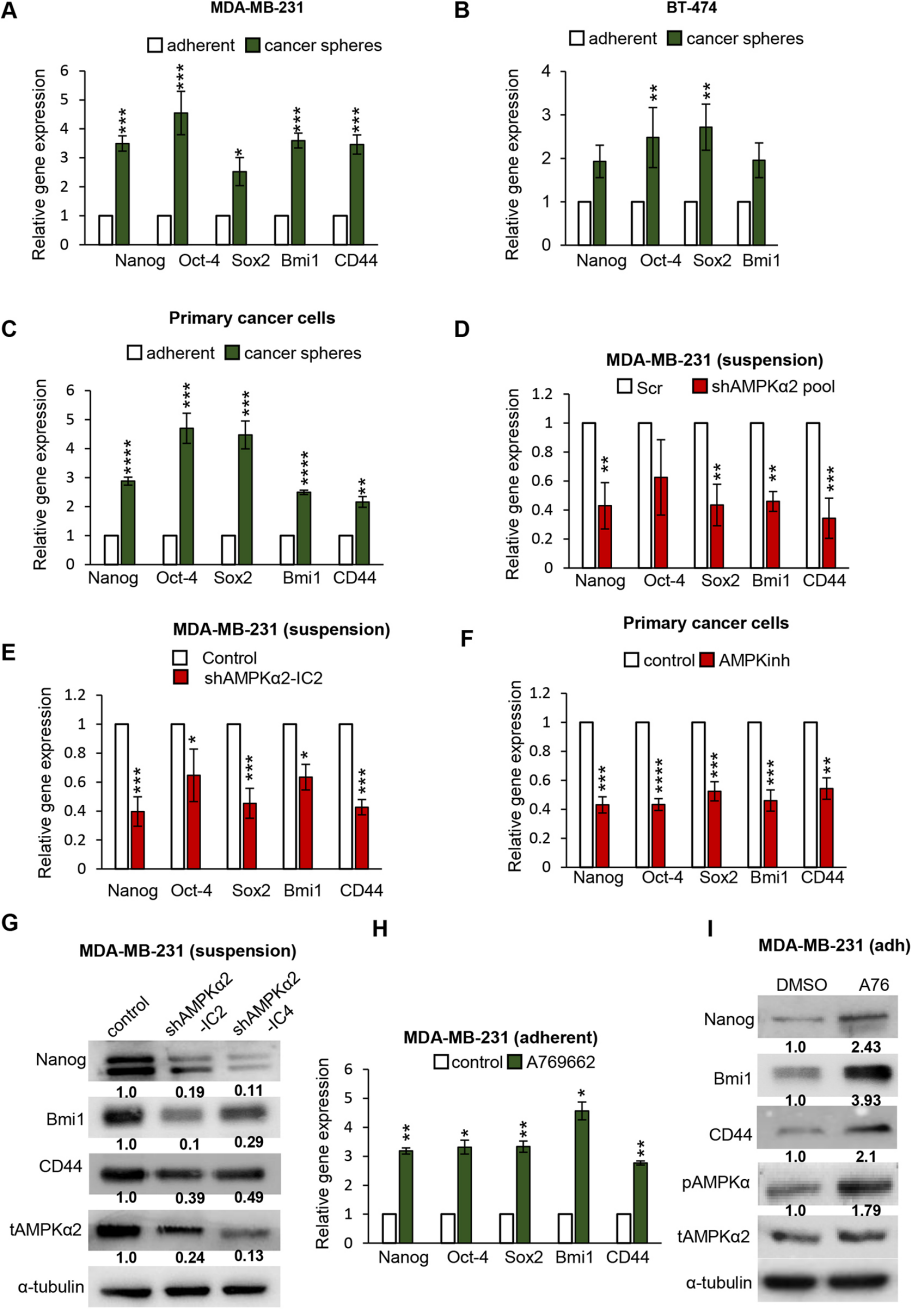
**AMPK enriches for BCSCs through transcriptional upregulation of stemness-related transcription factors.** (A,B) MDA-MB-231 (A) and BT-474 (B) were seeded in adherent and suspension. After 7 days, cells were harvested and subjected to qRT-PCR analysis for specified primer sets. *n*=3. (C) Primary breast cancer-derived cells were cultured in an adherent condition or as cancer spheres for 1 week in methyl cellulose, and were then harvested and subjected to qRT-PCR analysis for specified primer sets. *n*=4. (D) MDA-MB-231 cells stably expressing shRNA against AMPKα2-pool or scrambled shRNA (Scr) were cultured in suspension for 72 h, harvested and subjected to qRT-PCR analysis for specified primer sets. *n*=3. (E) MDA-MB-231 cells stably expressing (inducible) shRNA against AMPKα2-IC2 or control shRNA cells were treated with doxycycline and cultured in suspension for 72 h, then harvested and subjected to qRT-PCR for specified primer sets. *n*=3. (F) Primary breast cancer-derived cells were cultured in suspension in the presence of AMPK inhibitor (Compound C) for 72 h, harvested and subjected to qRT-PCR analysis for specified primer sets. *n*=4. (G) MDA-MB-231 cells stably expressing (inducible) shRNA against AMPKα2 (IC2 or IC4) or control shRNA cells were treated with doxycycline and cultured in suspension for 72 h, then were harvested and subjected to western blot analysis for specified antibodies. *n*=3. (H) MDA-MB-231 cells were cultured in an adherent condition in the presence of AMPK activator (A76) for 72 h, and were then were harvested and subjected to qRT-PCR analysis for specified primer sets. DMSO served as vehicle control. *n*=3. (I) Adherent MDA-MB-231 cells were cultured for 72 h in the presence of AMPK activator (A76), and were then harvested and subjected to western blot analysis for specified antibodies. DMSO served as vehicle control. *n*=3. Data are mean±s.e.m. *P<0.05, **P<0.01, ***P<0.001, ****P<0.001 (two-way ANOVA was performed for statistical significance).

In order to characterize the role of AMPK in BCSC self-renewal, we undertook serial sphere formation assays. A previous study from our laboratory showed that AMPKα2 (also known as PRKAA2) is predominantly expressed in several breast cell lines (Hindupur et al., 2014), and its knockdown reduces the phosphorylation of the bonafide substrate of AMPK and ACC substantially (Hindupur et al., 2014), suggesting that AMPKα2 is the predominant functional subunit. Therefore, we used multiple short hairpin (sh)RNA constructs to mediate the stable depletion of AMPKα2 in the invasive breast cancer cell lines MDA-MB-231 and BT-474. Using a shAMPKα2 pool knockdown approach (see Materials and Methods), we confirmed the reduction in AMPKα2 catalytic subunit in these cell lines compared to scrambled control (Fig. S1A). Depletion of AMPK significantly reduced the number of spheres under serial suspension culture in both the cell lines MDA-MB-231 and BT-474 (Fig. 1A,B). Representative phase-contrast images of the tumour spheres (Fig. S1B,C) show a drastic reduction in the number, as well as size, of the spheres. In addition, we used two independent AMPKα2-targeting inducible shRNA-expressing MDA-MB-231 stable cell lines (Saha et al., 2018). We have confirmed that these independent shRNA constructs also show more than a 75% reduction in AMPKα2 (Fig. 2G). Similar to the pooled shRNA data, AMPK depletion with the two independent shRNA constructs (shAMPKα2-IC2 and shAMPKα2-IC4) significantly reduced sphere formation capacity in these cell lines (Fig. S1D). *In vitro* serial sphere-forming potential serves as a surrogate for self-renewal and *in vivo* tumour initiation potential (Rajasekhar et al., 2011), and tumour-initiating cells can also be enumerated using CD44^high^/CD24^low^ marker profile (Al-Hajj et al., 2003) and high ALDH activity (Clark and Palle, 2016). We find that in MDA-MB-231 cells cultured in suspension for 72 h, AMPK depletion significantly reduced the number of CD44^high^/CD24^low^ BCSCs (Fig. 1C), as well as ALDH^high^ BCSCs (Fig. 1D). Independent inducible shRNA sequences targeting AMPK yielded similar results (Fig. S1E,F). Further, as the BT-474 cell line does not express CD44 (Olsson et al., 2011), we have additionally evaluated the CD44^high^/CD24^low^ marker profile-based assessment of stemness in MCF7 cells. AMPK inhibition using Compound C reduced the CD44^high^/CD24^low^ population in matrix-deprived MCF-7 cells (Fig. S1G). Together, these results suggest that AMPK promotes the self-renewal and maintenance of BCSCs.

As one of the major characteristics of cancer stem-like cells is their ability to initiate tumours *in vivo*, we examined the effect of AMPK knockdown on tumour initiation by performing limiting dilution experiments. The tumour engraftment in AMPK-depleted MDA-MB-231 cells was compromised upon serial dilution of injected cells compared to control scrambled shRNA-expressing cells, as demonstrated using an extreme limiting dilution assay (ELDA; Hu and Smyth, 2009) analysis (Fig. 1E). Tumour weights were also significantly lower upon AMPK knockdown (Fig. 1G; Fig. S1H). As the gold standard measure of stemness is the ability of cells to sustain long-term clonogenic potential upon serial transplantation in mice, we isolated tumour-derived cells from both scrambled and AMPK-depleted mice tumours, and re-injected them into new recipient mice. Confirming our earlier observations, tumour-derived cell transplantation caused much lower tumour initiation in the case of AMPK depletion compared to scrambled cells (Fig. 1F), which showed a statistically significant difference with ELDA analysis. Importantly, we performed assays to enumerate the number of stem cells in these mice tumour-derived cells. Using both CD44^high^/CD24^low^ marker profile (Fig. 1H) and ALDH^high^ activity (Fig. 1I), we observed that AMPK knockdown reduces the BCSC population *in vivo.* This parallels our results from the *in vitro* suspension cultured cells, together suggesting that AMPK contributes to the self-renewal of BCSCs in the tumour microenvironment.

Using yet another cell type, BT-474, we confirmed that AMPK depletion significantly reduced tumour initiation (Fig. 1J). Further, we observed that upon serial transplantation *in vivo*, AMPK-depleted BT-474 cells showed reduced capacity for tumour initiation (Fig. 1K). In addition, mice tumour-derived BT-474 cells also displayed a reduction in tumour weights (Fig. S1I). Additionally, similar to MDA-MB-231 tumour-derived cells, tumour-derived cells from BT-474 xenografts reduced the ALDH^high^ cells (Fig. S1J) in AMPKα2 knockdown cells. Independent shRNA sequences (IC-2 and IC4) targeting AMPKα2 also showed reduced tumour growth compared to control (Fig. S1K,L). Taken together, these data suggest that AMPK is a positive regulator of stemness in breast cancers.

### AMPK enriches for BCSCs through transcriptional upregulation of stemness-related transcription factors

As we have seen that the role of AMPK in increasing stemness *in vivo* can be modelled using *in vitro* cancer stemness-enriching suspension cultures, we used this system to query the mechanism of AMPK action. Increased stemness in suspension culture has been reported for prostate and human non-small cell lung cancers (Fan et al., 2012; Zhao et al., 2015). Our previously published data also show that compared to adherent breast cancer cells, suspension cultures promote stemness, as shown with real-time quantitative PCR (qRT-PCR) for *BMI1*, *NANOG* and *CD44* (Paranjape et al., 2012). Yet, these effects can be cell-type specific (Calvet et al., 2014; Rahman et al., 2015). Therefore, we set out to confirm this in our set of cells by performing various experiments between adherent and suspension cultures of both MDA-MB-231 and BT-474 cells. Indeed, compared to adherent cultures of MDA-MB-231 and BT-474, suspension increases the expression of multiple stemness markers, as seen with qRT-PCR (Fig. 2A,B) and western blotting (Fig. S2A). In addition, we also observed an increase in the CD44^high^/24^low^ population, as well as the ALDH^high^ population, in suspension in these cell lines (Fig. S2B). Additionally, these results hold true in primary patient-derived breast cancer cells (Fig. 2C), together suggesting that suspension promotes stemness properties in these breast cancer cells *in vitro*.

We reasoned that AMPK-dependent increase in stemness, seen functionally *in vivo* (Fig. 1), could occur by way of an increase in the expression of stemness-related transcription factors. As suspension culture enriches for stem-like cells, as seen previously (Fig. 2A-C; Fig. S2A,B), we used a suspension system for AMPK inhibition/depletion experiments, as these culture conditions allow a greater dynamic range for assessing the transcriptional effects of AMPK inhibition. We performed qRT-PCR for the major stemness-related transcription factors, such as *NANOG*, *SOX2*, *OCT4* and *BMI1*, as well as the marker *CD44*, a panel that predicts a transcriptional shift to a stem-like state in breast cancer cells. The shRNA-mediated AMPK depletion in stemness-enriching suspension cultures was significant in *NANOG*, *SOX2*, *BMI1* and *CD44* transcripts (Fig. 2D,E), and *NANOG*, Bmi1 and CD44 protein (Fig. 2G). The pharmacological inhibition of AMPK using Compound C in suspension-cultured MDA-MB-231 cells also caused a reduction of all genes in our stemness panel at transcript (Fig. S2C) and protein levels (Fig. S2F). This was also consistently seen in primary patient-derived breast cancer cells with qRT-PCR (Fig. 2F) and western blotting (Fig. S2F).

To further corroborate our observations of AMPK-triggered stemness in suspension that promotes AMPK activation, we undertook pharmacological activation of AMPK in adherent cultures using the specific AMPK activator A769662 (Göransson et al., 2007). The activation of AMPK in adherent cells consistently increased the transcriptional expression of the entire panel of stemness-related genes studied (Fig. 2H). Nanog, Bmi1 and CD44 protein levels were upregulated upon AMPK activation (Fig. 2I). This suggested that AMPK drives a transcriptional switch to a stem-like state.

Similar experiments extended to BT-474 showed that AMPK inhibition using pharmacological (Compound C) and RNAi methods in suspension cultures correspondingly reduced stemness markers at transcript levels, as seen with qRT-PCR (Fig. S2D,E), and protein levels (Fig. S2F). Significantly, AMP-depleted tumour-derived cells obtained after BT-474 injection into mice showed a reduction in *NANOG*, *OCT4* and *BMI1* gene expression, providing evidence that AMPK can also drive transcriptional upregulation and maintenance of stemness markers in the *in vivo* tumour microenvironment (Fig. S2G). As with MDA-MB-231, AMPK activation in attached BT-474 cells also increased stemness markers (Fig. S2H).

Together, we conclude that AMPK activation coordinates a stemness transcriptional response that causes an increase in the self-renewal capacity of BCSCs.

### AMPK drives stemness through Twist

**Fig. 3.**
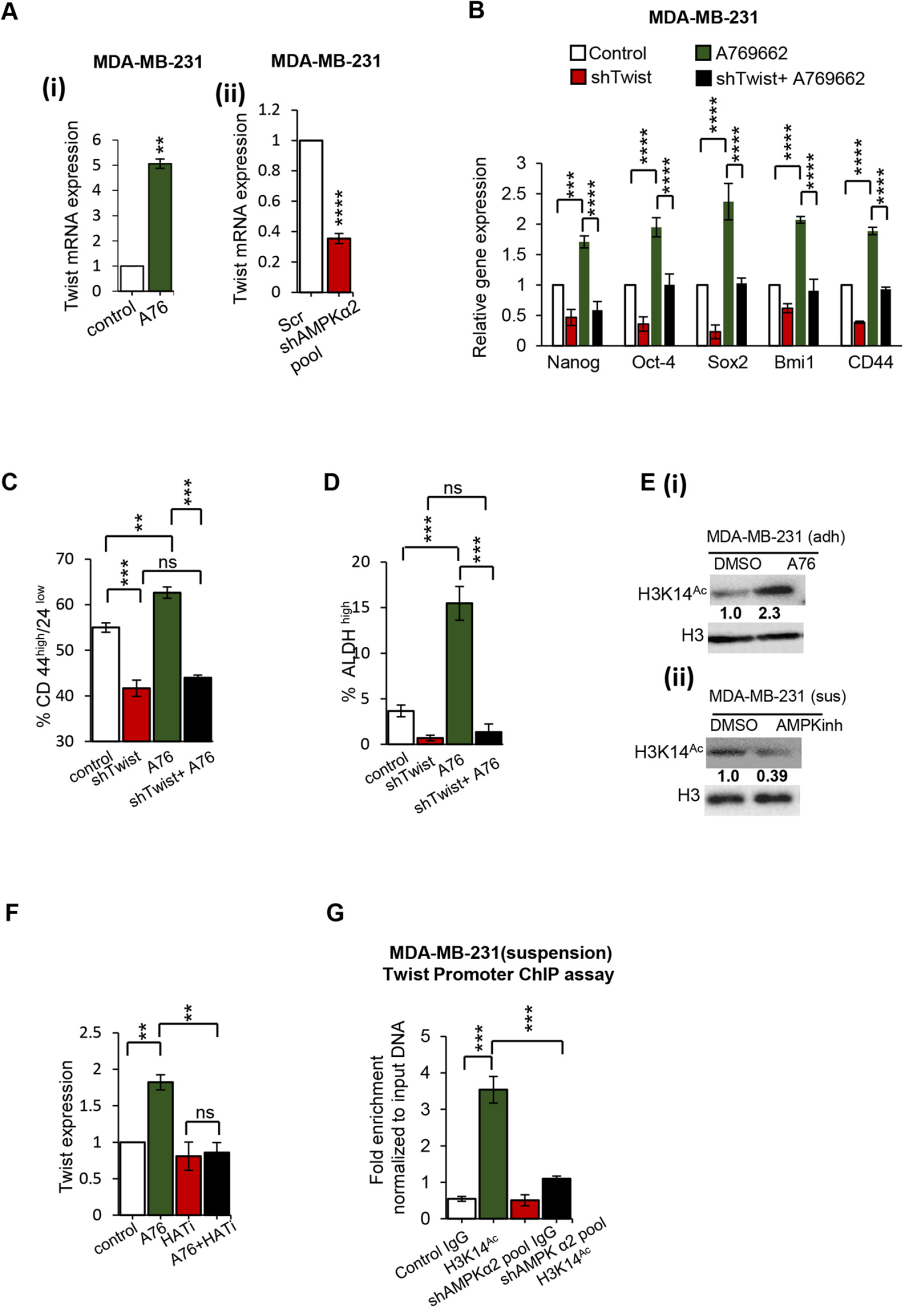
**AMPK drives stemness through TWIST.** (A) Adherent MDA-MB-231 cells were treated with AMPK activator for 72 h (Ai) and MDA-MB-231 cells stably expressing shRNA against AMPKα2-pool or scrambled shRNA (Scr) were cultured (Aii) for 72 h in suspension and then harvested and subjected to qRT-PCR for Twist. *n*=4. (B-D) Adherent MDA-MB-231 cells stably expressing shRNA against Twist or GFP cells (control) were cultured for 72 h in the presence of AMPK activator (A76). Cells were harvested and subjected to qRT-PCR (specified primer sets) (B), CD44^high^/24^low^ analysis (C) and an ALDH assay (D). *n*=4. (E) Adherent MDA-MB-231 cells were cultured for 72 h in the presence of AMPK activator (A76) (Ei) or AMPK inhibitor (Compound C) (Eii), and were then harvested and subjected to western blot analysis for specified antibodies. DMSO served as a vehicle control. *n*=3. (F) Adherent MDA-MB-231 cells were cultured for 60 h in the presence of AMPK activator (A76). After 60 h, cells were treated with HAT inhibitor for 12 h, and then harvested and subjected to qRT-PCR analysis for specified primer sets. DMSO served as a vehicle control. *n*=3. (G) MDA-MB-231 cells stably expressing shRNA against AMPKα2-pool or scrambled shRNA (Scr) were cultured in suspension for 72 h, harvested and subjected to a ChIP assay using H3K14ac antibody. IgG or beads alone served as negative controls for pulldown; input DNA served as positive control for qPCR. qRT-PCR analysis was carried out for the indicated primer sets (Twist promoter primers). Data are mean±s.e.m. **P<0.01; ***P<0.001; ****P<0.001; ns, not significant (two-way ANOVA or unpaired two-tailed Student's *t*-test).

Twist is a well-known transcription factor that regulates the process of EMT. Several recent reports suggest that Twist could drive cancer cell stemness (Vesuna et al., 2009; Yin et al., 2010). Previous work from our laboratory showed that AMPK stabilizes Twist protein to promote EMT (Saxena et al., 2018). As we found that AMPK drives an increase in stemness-related transcription factors, we investigated whether this transcriptional response was Twist dependent. Interestingly, we observed that AMPK activation in adherent cells caused an upregulation of *TWIST* expression at the transcript level in MDA-MB-231 (Fig. 3Ai), as well as in BT-474 (Fig. S3A). Correspondingly, AMPK depletion reduced *TWIST* gene expression in both MDA-MB-231 and BT-474 cells (Fig. 3Aii; Fig. S3A). We generated cells stably expressing shRNA against Twist to achieve a substantial depletion of Twist (Fig. S3B). Further, we activated AMPK using a specific pharmacological activator (A769662) in these cells lacking Twist. We found that AMPK-driven transcriptional upregulation of stemness factors was abrogated upon Twist depletion (Fig. 3B). Similarly, the population of BCSCs assayed using CD44^high^/CD24^low^ marker profile (Fig. 3C), as well as ALDH^high^ status (Fig. 3D), was also reduced upon Twist depletion. Additionally, Twist-depleted cells could no longer show an increase of stemness upon AMPK pathway activation (Fig. 3C,D), suggesting that AMPK drives the expression of stemness markers primarily through Twist.

Having established that the AMPK-Twist axis drives stemness in breast cancer, we next attempted to understand how AMPK regulates *TWIST* at the transcript level. AMPK is known to translocate to the nucleus, where it can phosphorylate histone H2B directly to coordinate stress-promoted transcription (Bungard et al., 2010). AMPK can also promote acetylation of the histones by inhibiting histone deacetylases through direct phosphorylation, as well as by altering the availability of the acetylation substrate acetyl CoA in the cells (Gongol et al., 2018). H3K14 acetylation is one of the hallmarks of transcriptional activation of a gene (Agalioti et al., 2002). We wanted to understand whether AMPK-driven *TWIST* transcription involves histone acetylation. First, we tested whether histone acetylation is globally altered upon AMPK modulation in our cell types of interest. Indeed, activation of AMPK with A769662 (Fig. 3Ei) increased H3K14 acetylation, and conversely, inhibition of AMPK activity (Fig. 3Eii) reduced the acetylation. Using histone acetyltransferase inhibitor C646 (HATI), we attempted to verify whether *TWIST* expression was specifically driven by histone acetylation. We observed that the AMPK-driven increase in *TWIST* transcription was completely abrogated in the presence of HAT inhibitor (Fig. 3F), suggesting that indeed AMPK regulates *TWIST* expression by altering the histone acetylation status. To prove that the activating histone H3K14 acetylation mark is indeed increased at the promoter of *TWIST* upon AMPK activation, we undertook chromatin immunoprecipitation (ChIP) assays. The primers were designed to detect the promoter or the coding region of *TWIST*, as indicated in the schematic in Fig. S3C.

In order to then address whether the H3K14 acetylation mark on Twist is AMPK dependent, we used cells expressing shRNA against AMPK. In stemness-enriching suspension cultures, although the control shRNA-expressing cells showed a robust H3K14 acetylation signal at the Twist promoter, AMPK depletion caused a significant reduction in the acetylation mark (Fig. 3G). This suggested that *TWIST* promoter acetylation is indeed primarily AMPK dependent. The acetylation mark was not detectable in the *TWIST* gene body sequences, as well as in yet another gene (*ABCG2*) promoter (Fig. S3D,E). Together, our results strongly suggest that AMPK drives *TWIST* expression through HAT-dependent H3K14 acetylation at the Twist promoter.

### AMPK contributes to chemotherapeutic drug resistance through Twist

**Fig. 4.**
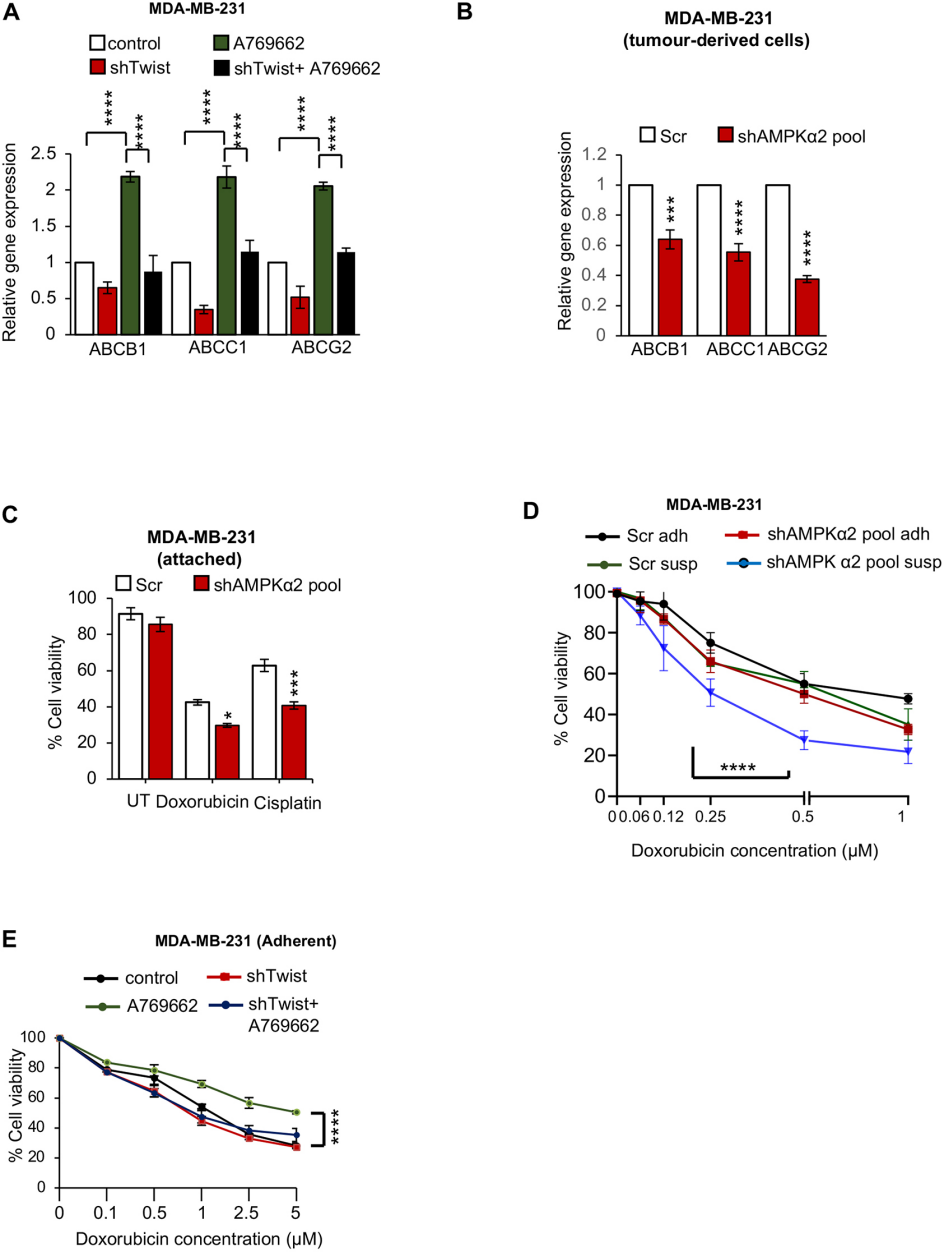
**AMPK contributes to chemotherapeutic drug resistance through Twist.** (A) Adherent MDA-MB-231 cells stably expressing shRNA against *TWIST* or GFP cells were cultured for 72 h in the presence of AMPK activator (A76), harvested and subjected to qRT-PCR (specified primer sets). *n*=4. (B) MDA-MB-231 cells stably expressing shRNA against AMPKα2-pool or scrambled shRNA (Scr) were injected (10^6^) subcutaneously into five female nude mice (Scr shRNA cells in the left flank, and shAMPKα2-pool cells in the right flank) after the specified time of tumour formation. Tumours were isolated and subjected to qRT-PCR analysis for specified primer sets. *n*=3. (C) Adherent MDA-MB-231 cells stably expressing shRNA against AMPKα2-pool or scrambled shRNA (Scr) were treated with various concentrations of doxorubicin or cisplatin for 48 h, and were subjected to a cell viability assay (MTT). *n*=4. (D) MDA-MB-231 cells stably expressing shRNA against AMPKα2-pool or scrambled shRNA (Scr) were cultured in adherent and suspension condition in the presence and absence of doxorubicin for 48 h, and were then subjected to a cell viability assay (MTT). *n*=4. (E) Adherent MDA-MB-231 cells stably expressing shRNA against Twist or GFP cells were treated with A76 or DMSO (DMSO served as a vehicle control) and various concentrations of doxorubicin for 48 h. Thereafter, cells were subjected to an MTT assay. *n*=3. Data are mean±s.e.m. ***P<0.001, ****P<0.001 (two-way ANOVA).

Twist is known to modulate ABC transporter expression during the process of EMT (Saxena et al., 2011). Stem-like cells are known to be drug resistant and one of the pathways deployed involves an increased expression of ABC transporters. Having seen that the AMPK-Twist axis can increase breast cancer stemness, we next investigated whether this axis was responsible for the chemotherapeutic drug resistance property of BCSCs. In MDA-MB-231 cells, expression of the three major ABC transporters *ABCB1*, *ABCC1* and *ABCG2* increased upon AMPK activation (Fig. 4A). Interestingly, this increase was abrogated in Twist-depleted cells (Fig. 4A). Importantly, mouse tumour-derived MDA-MB-231 cells (pooled shRNA and independent shRNA stable cells) with AMPK depletion had a reduced level of all the three ABC transporter types studied (Fig. 4B; Fig. S4A). We strengthened these results by replicating the above approach in the BT-474 cell line. Depletion of AMPK reduced ABC transporter expression not only *in vitro* (Fig. S4B) but also in tumour-derived cells from mice (Fig. S4C), showing that the regulation is robust enough to persist in the *in vivo* cancer microenvironment. We wanted to assess whether AMPK-dependent ABC transporter expression actually contributed to increased drug resistance in cells. The treatment of adherent MDA-MB-231 cells with doxorubicin and cisplatin revealed that AMPK depletion makes cells more sensitive to these drugs, as seen by the reduction in cell viability (Fig. 4C). Similar results were obtained with BT-474 (Fig. S4D). Thus, the increase in transporter expression correlates with drug resistance to commonly used chemotherapeutic drugs.

We then tested the effect of varying concentrations of doxorubicin in adherent and suspension cultures of MDA-MB-231 cells, and queried whether AMPK depletion is likely to have a significant effect on the drug-resistance phenotype in stemness-enriching suspension cultures. In keeping with the context-dependent role of AMPK in matrix-deprived cells, depletion of AMPK drastically reduced the half-maximal inhibitory concentration (IC_50_) of suspended MDA-MB-231 cells for doxorubicin from 0.6 µM to 0.3 µM (Fig. 4D). Although adherent cultures of MDA-MB-231 were only partly dependent on AMPK for their chemoresistance, as seen in Fig. 4C,D, in stemness-enriching suspension cultures, MDA-MB-231 cells activated AMPK and relied on this stress kinase for chemoresistance to doxorubicin. This was also evident in doxorubicin-treated MDAMB231 cells expressing two independent inducible AMPK shRNA sequences (Fig. S4E). Thus, the increase in the ABC family of transporters through AMPK activation has a functional correlation in the form of increased chemoresistance, especially in suspension cultures.

In order to clarify the role of the AMPK-Twist axis in drug resistance, we used the Twist-depleted MDA-MB-231 cells and measured cell viability under various concentrations of doxorubicin. Although activation of AMPK alone improved cell viability, simultaneous Twist depletion abrogated this AMPK-driven drug-resistance phenotype (Fig. 4E). These results are consistent with Twist being responsible for both ABC transporter expression and chemoresistance downstream of AMPK. These results, taken together, suggest that the AMPK-Twist axis is not only crucial for stemness but also for the drug-resistance phenotype of the BCSCs.

### Post chemotherapy patient samples display an increase in the AMPK-Twist axis

Pre-operative or neoadjuvant therapy is often used in aggressive breast cancers to achieve optimal surgical resection. It was reported previously that chemotherapy could induce breast cancer stemness (Saxena et al., 2011). We wanted to understand whether the AMPK-Twist axis contributes to the increased stemness and drug resistance *in vivo*.

**Fig. 5.**
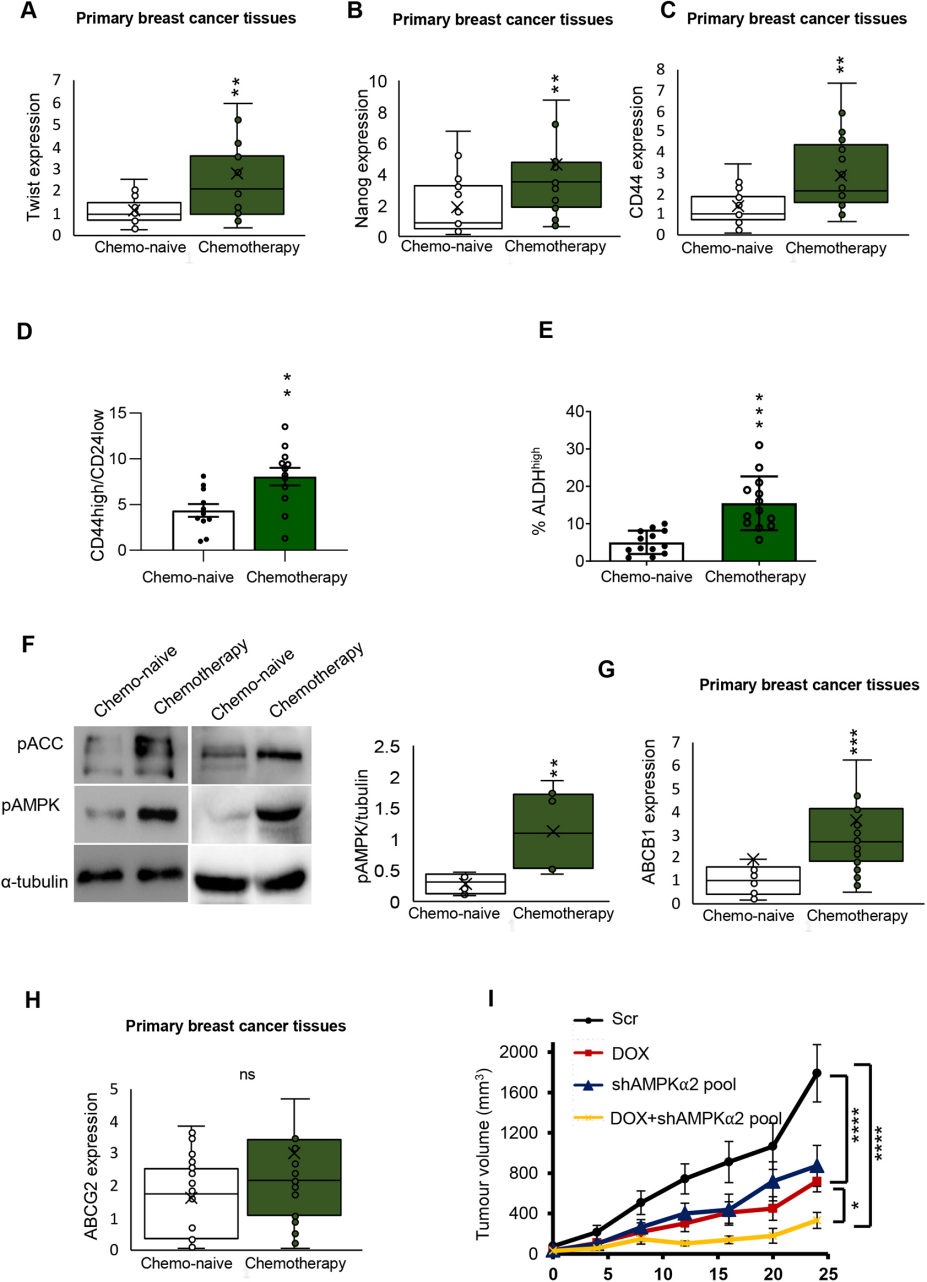
**Drug-resistant post chemotherapy patient samples display an increase in the AMPK-*TWIST* axis.** (A) qRT-PCR analysis of *TWIST* expression in chemonaive (*n*=33) and chemotherapy-treated patient tissue samples (*n*=21). (B,C) qRT-PCR analysis of *NANOG* (B) or *CD44* (C) mRNA expression in chemonaive (*n*=21) and chemotherapy-treated (*n*=21) patient tissue samples. (D,E) Chemonaive (*n*=21) and chemotherapy-treated (*n*=21) patient tissue samples were collected, and cells were isolated and subjected to CD44^high^/24^low^ analysis (D) and ALDH assay analysis (E). *n*=3. (F) Chemonaive and chemotherapy-treated patient tissue samples were collected and subjected to western blot analysis for pACC and pAMPK. Blots were quantified using image J (*n*=6). (G,H) qRT-PCR analysis of *ABCB1* (G) and *ABCG2* (H) mRNA expression in chemonaive (*n*=21) and chemotherapy-treated (*n*=21) patient tissue samples. (I) BT-474 cells stably expressing shRNA against AMPKα2-pool (10^6^) or scrambled shRNA (Scr) (5×10^5^) were subcutaneously injected into ten female nude mice (Scr shRNA cells in the left flank, and shAMPKα2 cells in the right flank). After primary tumour formation (∼100 mm^3^), mice were randomized into two groups (five mice/group) and treated with doxorubicin (DOX) or vehicle control every 4 days for 4 weeks. Tumour kinetics were measured for a specific period of time. Relative quantification of mRNA expression in A-E,G,H was calculated using β2 M as housekeeping gene. Data are mean±s.e.m. *P<0.05; **P<0.01; ***P<0.001; ****P<0.001; ns, not significant (two-way ANOVA).

We first queried whether, compared to chemonaive tissues, post chemotherapy tissues obtained from patients after neo-adjuvant treatment with doxorubicin and methotrexate had increased expression of stemness markers. Indeed we found a significant increase in *TWIST* gene expression (Fig. 5A) in post chemotherapy samples. A concomitant increase in the *TWIST* target genes *NANOG* and *CD44* (Deng et al., 2016) was also detected after chemotherapy in the same set of samples (Fig. 5B,C). In addition, we performed CD44^high^/24^low^ and ALDH^high^ subpopulation analysis using chemonaive and chemo-treated patient samples. Our analysis revealed that chemotherapy significantly increased the CD44^high^/CD24^low^ and ALDH^high^ population compared to chemonaive samples (Fig. 5D,E). We also measured AMPK activation in some of these samples using western blotting for phosphorylated (p)AMPK and pACC levels. A significant increase in these phosphorylations indicated that AMPK signalling is upregulated upon chemotherapeutic treatment (Fig. 5F). *ABCB1* but not *ABCG2* expression was significantly increased under neoadjuvant treatment (Fig. 5G,H). Together, our results reveal that the AMPK-Twist pathway is critical for maintaining BCSCs, and contributes to chemotherapeutic resistance of breast tumours in patient-derived breast cancer samples.

### Combinatorial regimes using AMPK inhibition with doxorubicin reduce tumour growth in mice

Having established thus far that AMPK regulates BCSC stemness and drug-resistance phenotypes, we wondered whether combinatorial targeting of this adaptive stress kinase, along with administration of the standard chemotherapeutic drug doxorubicin, would benefit breast cancer patients. We have previously seen that combining AMPK depletion with doxorubicin causes a reduction in the cell viability of MDA-MB-231 cells in stemness-enriching suspension culture (Fig. 4D). We wanted to explore whether this would be true in the *in vivo* mouse models. To address this, we used two different approaches to inhibit AMPK in mouse tumours. We injected 10^6^ cells of the BT-474 cell line, which robustly forms tumours, into each flank of the mouse. Treatment regimens were started when the tumour was 100 mm^3^. We monitored tumour volumes after AMPK inhibitor Compound C (2 mg/kg/4 days) or doxorubicin (4 mg/kg/week) treatment, alone or in combination. Tumour growth was reduced following individual treatments but the combined treatment was significantly better at reducing tumour growth (Fig. S5A,B), suggesting that this drug regimen possibly targets both the bulk and stem-like cells to prevent tumour growth. The tumours from each of these conditions were paraffin embedded and immunohistochemistry (IHC) was performed for two stemness markers, Oct4 and Bmi1 (Fig. S5C). The combinatorial regime of doxorubicin with AMPK inhibition drastically reduced stemness marker expression *in vivo*. Semiquantitative intensity analysis of the IHC sections (Fig. S5D,E) showed a significant reduction in Oct4 and Bmi1 expression in the combinatorial treatment, and doxorubicin alone enriched for stemness markers after eliminating the bulk tumour cells. Further, we assessed the stem cell population using an ALDH assay with tumour-derived cells. Quantitative fluorescence-activated cell sorting (FACS) analysis showed a reduction of ALDH^high^ cells with combinatorial therapy and Compound C alone, whereas doxorubicine alone enriched the stem cell population (Fig. S5F). These results suggest that combinatorial therapy with AMPK inhibitors alongside standard chemotherapeutic drugs could benefit breast cancer patients. In order to confirm these observations, we used an alternative approach to target AMPK using stable expression of shRNA. As seen previously, shAMPKα2 pool cells form much smaller tumours. To start doxorubicin treatment at comparable tumour volumes, we injected double the number of shAMPKα2-pool-expressing cells. As with pharmacological inhibition, RNAi-mediated AMPK knockdown also enhanced the efficacy of doxorubicin in reducing tumour growth (Fig. 5I). Together, these results suggest that targeting of AMPK could inhibit the acquisition of the BCSC phenotype, thereby making the tumours more chemosensitive.

## DISCUSSION

The AMPK pathway is a context-dependent signal that promotes or inhibits tumour progression. Although more traditionally known for its tumour-suppressive actions, owing mainly to its growth-halting effect through the inhibition of mTOR signalling and stabilisation of p53, emerging studies have begun to highlight contextual oncogenic roles for AMPK. Our previous work has elucidated a role for this pathway in regulating metabolic phenotype, especially in the circulating anchorage-deprived cancer cells (Saha et al., 2018). Therefore, the role of this pathway depends on the cancer tissue type and the micro-environmental cues. Several studies have suggested that AMPK inhibits stemness. For example, microRNA 448 targets MAGEA6, a negative regulator of AMPK, thereby activating the pathway and suppressing stemness in hepatocellular carcinoma (Guo et al., 2019). Similarly, AMPK promotes Nanog degradation to inhibit self-renewal and chemoresistance of prostate cancer cells (Wang et al., 2019). On the other hand, extracellular stress signals, such as glucose deprivation, cause AMPK activation and nuclear translocation, along with the promotion by PKM2 of Oct4-dependent expression of cancer stemness genes in pancreatic adenocarcinoma cells (Yang et al., 2018). Our current study sheds light on AMPK as a novel positive regulator of breast cancer stemness and drug resistance. We have demonstrated that the inhibition of AMPK depletes the BCSC population and increases chemosensitivity. Further, in the context of hypoxia, a study undertaken by our laboratory demonstrated that AMPK promotes breast cancer stemness by increasing Notch1 stability (Lahiry et al., 2020 preprint). Our results on the BCSCs, taken together, support the idea that the presence of extracellular stress signals, such as nutrient or anchorage deprivation and hypoxia, can drive an AMPK-dependent adaptive state transition to a stem-like phenotype. This highlights a novel context-dependent role of AMPK in maintaining breast cancer stemness.

AMPK and mTOR signalling are considered master regulators of cell metabolism. They are interlinked and opposing signalling pathways (González et al., 2020). Restrained mTOR activity has been previously reported to be required for self-renewal and differentiation of mammary stem cells (Mohapatra et al., 2017). We and others have previously shown that AMPK activation leads to inhibiton of mTOR signalling in suspension (Saha et al., 2018). Thus, our study supports a model for active AMPK signalling with concomitant loss of mTOR signalling in breast cancer stemness. However, the direct role of mTOR in this context needs to be explored further. Our data show a complete loss of AMPK-driven increase in stemness upon knockdown of Twist. Therefore, our data suggest that Twist is one of the prime downstream effectors of AMPK-driven stemness.

Although the context dependence of AMPK in cancer cell survival is now appreciated, we provide evidence that this also extends to properties such as stemness and drug resistance. This is a critical insight that now allows us to combine standard chemotherapeutic drugs, such as doxorubicin and cisplatin, with AMPK inhibitors to reduce the stemness of breast cancers. Given that anchorage deprivation, hypoxia and other micro-environmental stress signals are likely coupled with cancer metastasis (Mori et al., 2009), our study proposes a more (patho)physiologically relevant role for AMPK that would not have come to light when adherent cells are studied. AMPK inhibition alongside doxorubicin can prevent state transition to a stem-like state, thereby enabling standard chemotherapeutic drugs to more effectively target tumourigenic cells. Critically, there is a need to develop more specific inhibitors for AMPK that can be used for cancer therapeutics.

## MATERIALS AND METHODS

### Collection of normal, tumour and chemo-treated breast tissue samples

Adjacent normal chemonaive tumour breast tissues and chemo-treated tumour tissue samples from grade III invasive breast ductal carcinomas were obtained from the Kidwai Memorial Institute of Oncology (KMIO) Bangalore, in accordance with the Institutional Review Board and in compliance with the ethical guidelines of KMIO and the Indian Institute of Science (IISc). This study was approved by the Institutional Human Ethics Committee, IISc, and patient consent was acquired in writing before surgery. Tissue samples details are featured in Table S1. Normal tissue was excised ∼6 cm away from the tumour and was confirmed by pathologists for the absence of tumour cells. As per the neo-adjuvant treatment protocol, patients were treated with 5-FU (5-flurouracil) and doxorubicin for three cycles (each cycle for 21 days). Surgically resected tumour tissue was collected and divided into two parts. One part was harvested for RT-PCR and the other part was used to isolate single cells for cell-based experiments. For RNA isolation, normal, chemonaive or chemo-treated tumour tissue chunks were collected in RNAlater (Qiagen, Hilden, Germany). For FACS analysis, single cells were used. For experiments involving live culture, tissues were minced with Dulbecco's modified Eagle/Nutrient Mixture F-12 medium containing collagenase and hyaluronidase, and minced tissue chunks were hybridized at 37°C for 12-14 h. Normal and tumour organoids were isolated by centrifugation at 200 ***g*** for 1 min. After 4 h, organoids were trypsinized, and single cells were isolated by passing through a 100-μn cell strainer and cultured in serum-free medium containing 10 ng/ml human epidermal growth factor, 1 µg/ml hydrocortisone, 10 µg/ml insulin, 4 ng/ml heparin and B27. Single cells were seeded in regular tissue-culture plates for adherent culture, or in ultra-low attachment plates (Corning) for mammosphere culture.

### Cell lines and cell culture conditions

Breast cancer cell lines MDA-MB-231, BT-474 and MCF-7 were procured from American Type Culture Collection, and validated by short tandem repeat analysis. These cell lines were cultured in Dulbecco's modified Eagle medium (DMEM, Sigma-Aldrich) supplemented with 10% fetal bovine serum (FBS) containing penicillin and streptomycin, at 37°C and 5% CO_2_. Cell lines were used for experiments within ten passages after thawing.

### Plasmids, transfection and generation of stable cell lines

#### Pool AMPK KD

AMPK α2 knockdown stable cells were generated by transfecting MDA-MB-231 and BT-474 cells with a pool of four shRNA constructs targeting AMPKα2 (pRFP-C-RS-PRKAA2sh1, 5′-TGTCTGCTGTGGATTACTGTCATAGGCAT-3′; pRFP-C-RS-PRKAA2sh2, 5′-TCTGGAGCTGTGGTGTTATCTTGTATGCT-3′; pRFP-C-RS-PRKAA2sh3, 5′-TCAAGACCAGCTTGCAGTGGCTTATCATC-3′; and pRFP-C-RS-PRKAA2sh4, 5′-GCTGGCTTACACAGACCAAGATCAAGTT-3′) (Origene Technologies, Rockville, MD, USA) using Lipofectamine 2000 (Invitrogen). Scrambled HuSH-29 shRNA (5′-GCACTACCAGAGCTAACTCAGATAGTACT-3′) vectors were used as controls. The generated knockdown cells using a pool of four shRNA constructs were named as AMPK α2 pool, and have been described previously (Saha et al., 2018).

#### Inducible AMPK KD

Inducible AMPKα2 knockdown stable cells were generated by transfecting MDA-MB-231 cells with two independent shRNA constructs targeting AMPKα2 [V2THS_57674 (shAMPK-IC2, 5′-TTAACTGCCACTTTATGGC-3′) and V3THS_375315 (shAMPK-IC4, 5′-TATTTTTCCAACAACATCT-3′)] procured from Dharmacon (Pittsburgh, PA, USA) as described previously (Saha et al., 2018); empty pTRIPZ vector was used as control.

Inducible shRNA against AMPKα2 (V2THS_57674) and the corresponding control empty pTRIPZ vector were procured from Dharmacon. Lipofectamine (Invitrogen) was used to transfect plasmid DNA into cells. Stable cells were generated using puromycin (0.5 μg/ml) selection followed by flow cytometer-based sorting (MoFlo; Beckman Coulter, Brea, CA, USA) for RFP expression (encoded by the vector), and were expanded and frozen for future use. Knockdown was confirmed by immunoblotting.

### Pharmacological chemical inhibitors and activators

Pharmacological chemicals used in this study include AMPK activator A769662 (100 μM, University of Dundee, UK) (Göransson et al., 2007) and AMPK inhibitor 6-[4-(2-piperidin-1-ylethoxy-phenyl)]-3-pyridin-4-yl-pyrrazolo [1,5-a]-pyrimidine (10 μM, Compound C, Calbiochem) (Kim et al., 2008).

### RNA isolation and cDNA synthesis

RNA isolation was carried out using TRIzol as described previously (Balaji et al., 2016). Briefly, RNA was isolated using the TRIzol-chloroform method, and total RNA was quantified using a nanodrop spectrophotometer. Prior to cDNA synthesis, total RNA was treated with DNase1 to eliminate genomic DNA. cDNA was synthesized from 2 μg of DNase-treated RNA using a high capacity cDNA reverse transcription kit (Applied Biosystems) according to the manufacturer's instructions. Primers for stemness markers, ABC family of transporters and housekeeping genes were designed using Primer 3 software. qRT-PCR was carried out using SYBR Green Master Mix with an ABI 7500 RT-PCR system, starting with 10-20 ng of cDNA, and analyzed using the Δct method with β2 M and HPRT as reference markers. Δct was determined by subtracting Δct of each test sample from the average Δct of control sample. The differences in mRNA expression of all the genes were calculated as the fold change using the formula 2-ΔΔct.

### Immunoblotting

Cells were harvested for the isolation of protein for western blot analysis using lysis buffer containing 1% NP40 detergent, 0.5% sodium deoxycholate, 0.1% SDS, 50 mM sodium fluoride, 1 mM sodium orthovanadate, 10 mM sodium pyrophosphate (Sigma-Aldrich) and protease inhibitors (Roche, Mannheim, Germany). Protein concentrations were estimated using Bradford reagent, and an equal amount of protein (100 μg) was resolved by SDS-PAGE using Bio-Rad apparatus, transferred to a polyvinylidene difluoride membrane (Millipore, Billerica, MA, USA) and probed with appropriate antibodies. α-Tubulin (Calbiochem) served as loading control. Horseradish peroxidase-coupled secondary antibodies were obtained from The Jackson Laboratory, and immunoblots were visualized using PICO reagent (Pierce, Waltham, MA, USA). Primary antibodies used were phospho AMPK (Thr172; #2535), total AMPK α2 (#2757), AMPK α(1/2) (#2535), phospho ACC (#11818), total ACC (recognizes both isoforms 1 and 2; #3676), Bmi1 (#6964), CD44 (#37259), H3K14^Ac^ (#MA5-24668) and H3 (#PA5-16183), which were obtained from Cell Signaling Technology (Beverly, MA, USA), and Nanog (sc-293121) and Oct-4 (sc-5279), which were obtained from Santa Cruz Biotechnology. All primary antibody dilutions were 1:1000 and secondary antibody dilutions were 1:5000.

### Sphere formation and self-renewal assays

Cells were treated with AMPK inhibitor, Compound C and DMSO as a vehicle control for 24 h. After incubation, cells were trypsinized and resuspended (10^5^ cells/ml) in 1.5% methyl cellulose (in DMEM supplemented with 10% FBS). Cells were seeded on 0.6% noble-agar-coated plates at a density of 10^5^ cells/35 mm dish. Treatment was continued for 9 days (with supplementation of medium) to score for total cancer spheres in each dish. Only those spheres that contained more than 20 cells were counted as spheres. After counting the primary cancer spheres, spheres were trypsinized and converted into single cells, and again cells (10^5^ cells/ml) were seeded for secondary cancer sphere formation without drug. The total number of spheres formed was counted in each case.

### Cell viability assay

Cells were seeded into a 96-well plate (8×10^3^ cells/well) and after 12 h, cells were treated with AMPK inhibitor (10 µM), anti-cancer agents, such as doxorubicin (1 µM) and cisplatin (20 µM), and DMSO (vehicle control), then further, cells were incubated for 48 h. After 48 h of incubation, 20 µl of 2,5-diphenyl-2H-tetrazolium bromide (MTT) reagent (5 mg/ml) was added to each well of the plate, followed by incubation for another 4 h. Then medium was removed and 100 µl of DMSO was added to each well to dissolve the formazan crystals, and absorbance was measured using a plate reader at 550 nM. IC_50_ values were calculated using the curve fit method and GraphPad Prism 5.

### ALDH activity assay

Single-cell suspensions of AMPK-depleted/inhibited cells (10^5^) were subjected to an ALDH assay, as per the manufacturer's instructions (Stemcell Technologies, USA). Briefly, cells were centrifuged at 300 ***g*** and the pellet was washed with PBS and resuspended in 1 ml of ALDH buffer containing ALDH substrate (1 µl). As a negative control for each sample, cells (10^5^) exposed to ALDH substrate were transferred to a diethylaminobenzaldehyde-containing tube to specifically inhibit ALDH activity. After incubation at 37°C for 30 min, cells were pelleted down at 300 ***g*** and resuspended in 400 µl of fresh ALDH buffer. ALDH activity was analyzed by ALDH^high^ on the *x*-axis and SSC^low^on the *y*-axis using BD FACS-Canto (BD Biosciences). Data were analysed using Summit 5.2 software. Representative FACS dot plots are shown in the Supplementary Materials and Methods.

### CD44^high^/24^low^ assay

Cells were trypsinized to obtain single-cell suspension and incubated at 37°C for 1 h to retrieve surface antigens. Cells were stained with CD44 antibody and CD24 antibody conjugated to PE-CY7 or PE or fluorescein isothiocyanate (FITC), and incubated on ice for 45 min with intermittent mixing every 15 min. After incubation, cells were washed twice with 1× PBS, and 10,000 events were analyzed per sample in a BD-FACS canto-flow cytometer equipped with excitation at 488 nm and emission at 785 nm for PE-CY7, excitation at 488 nm and emission at 578 nm filters for PE, and excitation at 488 nm and emission at 525 nm filters for FITC. Data were analysed using Summit 5.2 software. Representative FACS dot plots are shown in the Supplementary Materials and Methods.

### Annexin-V assay

For apoptosis analysis, MDA-MB-231 cells stably expressing (inducible) shRNA against AMPKα2 (IC2 or IC4) and control cells were seeded into a six-well plate. Cells were induced with doxycycline for 48 h and then treated with doxorubicin (1 μM) for 20 h. Cells were then trypsinized, counted and incubated with Annexin-V-FITC in Annexin-V binding buffer (BD Biosciences). After 15 min of incubation, cells were analyzed and data were analysed using Summit 5.2 software.

### Serial dilution and serial transplantation experiments *in vivo*

MDA-MB-231 or BT-474 cells stably expressing shAMPKα2-pool or scrambled sequence were trypsinized, counted and subcutaneously injected (10^5^, 10^5^ and 10^6^) into each flank of the 15 mice (for MDA-MB-231) and 20 female athymic nude mice (for BT-474). Scrambled shRNA (Scr) cells were injected into the left flank of the mice, and shAMPKα2-pool cells were injected into the right flank of the five mice for each cell dilution. Tumour sizes were measured for every 7 days until the specified time (60 days). ELDA analysis was performed using 42 days (MDA-MB-231) and 35 days (BT-474) tumour kinetics data. After the specified time, tumours were surgically isolated and weighed. Tumours (isolated from 10^6^ injected set of mice) were homogenized and harvested for western blot analysis, and other tumours were digested with collagenase and trypsinized for *in vitro* culture. These tumour cells from mice were cultured and stained for CD44^high^ and CD24^low^ analysis, and an ALDH activity assay. Remaining cells were counted and injected into another set of athymic nude mice. MDA-MB-231 (10^5^ and 10^5^) or BT-474 (10^5^ and 10^4^) tumour-derived scrambled cells were injected into the left flank of the mice, and shAMPKα2 cells were injected into the right flank of the mice. Tumour size was measured every 7 days for 42 days for MDA-MB-231, and tumour size was measured every 5 days for 35 days for BT-474 cells. Extreme limiting dilution was performed for statistical analysis.

### Xenograft assay using inducible shAMPKα2 cells

MDA-MB-231 cells stably expressing (inducible) shRNA against AMPKα2 (IC2) were injected into the left flank of the mice (five mice), and control cells were injected into the right flank of the mice. Similarly, shAMPKα2 (IC4) cells were injected into the left flank of the mice (five mice) and control cells were injected into the right flank. After tumours reached 100 mm^3^ in size, mice were treated with doxycycline for a period of 30 days. During the treatment period, tumour sizes were measured for every 5 days, and after the specified time, tumours were surgically isolated and weighed.

### Xenograft assay using BT-474 cells

BT-474 cells were trypsinized and counted (10^6^/100 µl), and were subcutaneously injected into each flank of the 20 female athymic nude mice. The mice were allowed to form tumours up to 100 mm^3^ in size Then, the mice were randomly divided into four groups with five mice in each. DMSO was injected intraperitoneally as a vehicle control and served as the control group. Compound C (2 mg/kg) was intraperitoneally injected into the second group for every 4 days, doxorubicin (4 mg/kg) was injected into the tail vein of the third group of mice every week, and the fourth group received dual treatment of doxorubicin and Compound C. Tumour volume was measured on every 4 days for 24 days. After 24 days, tumours were excised and tumour images were captured.

### Xenograft assay with shAMPKα2 cells and treatment with doxorubicin

BT-474 cells stably expressing shRNA against AMPKα2 or scrambled shRNA (control) cells were injected subcutaneously into ten female nude mice [scrambled cells in the left flank (10^5^), and shAMPKα2-pool cells (10^6^) in the right flank]. After primary tumour formation (∼100 mm^3^), mice were randomized into two groups (five mice/group) and treated with doxorubicin or vehicle control for every 4 days for 4 weeks. Tumour kinetics were measured for a specific period of time for every 5 days.

### IHC and tissue samples

Mouse tumours were isolated from the control and treated mice. Paraffin-embedded sections were deparaffinized using xylene, and rehydrated with alcohol. Then the sections were incubated for 15 min in a mixture of 5% hydrogen peroxide and 95% methanol to quench the peroxidase activity. Antigen retrieval was carried out by revealing the sections to steam at high pressure in a conventional pressure cooker by placing the sections in 10 mM of freshly prepared sodium citrate buffer (pH 6). After antigen retrieval, sections were blocked with 4% skimmed milk powder for 1 h to avoid non-specificity. Primary antibodies (Bmi1 and Oct4) were diluted (according to the manufacturer's instructions) in skimmed milk powder, and the sections were incubated with primary antibodies at 4°C overnight. The secondary anti-mouse and anti-rabbit antibodies, tertiary antibody and DAB kit were procured from Bangalore Genei, Bangalore, India. Haematoxylin and Eosin staining was carried out separately for these tumours.

### ChIP analysis

A ChIP assay was carried out essentially as per the standard protocols (Boyd and Farnham, 1999). In brief, cells were harvested after 72 h of suspension, crosslinked with 1% formaldehyde for 10 min at room temperature, and then sonicated to obtain chromatin fragments ranging from 300 bp to 700 bp. After pre-clearing with blocked protein-A beads (Bangalore Genei), the soluble chromatin was equally divided and immunoprecipitated with a rabbit anti-IgG antibody (isotype control; Bangalore Genei) and incubation at 4°C overnight. A ‘no antibody’ control was also incubated at 4°C. Following washes, the antibody-protein-DNA complex was eluted from the beads, followed by reverse crosslinking at 65°C with 200 mM NaCl. After RNase (Sigma-Aldrich) and proteinase K (Sigma-Aldrich) treatment, the DNA was purified by phenol-chloroform extraction. An equal amount of DNA was subjected to qPCR with primers specific for Twist promoter and the gene body.

### Statistical analysis

Statistical significance was determined using unpaired two-tailed Student's *t*-test, ANOVA and two-way ANOVA. The curve-fit method was used to analyse the IC50 value. GraphPad Prism version 5 was used for all statistical tests and Excel was used for plotting the graphs. Data are shown as mean±s.e.m. (*P<0.05, **P<0.01, ***P<0.001, ****P<0.001).

## Supplementary Material

Supplementary information
