# Supplementary material for: AMP-activated protein kinase promotes breast cancer stemness and drug resistance
Source: Dis Model Mech. 2022 May 27;15(6):dmm049203. doi: 10.1242/dmm.049203 (PMC9150117; doi:10.1242/dmm.049203)
Supplement: Supplementary information [file dmm-15-049203-s1.pdf]

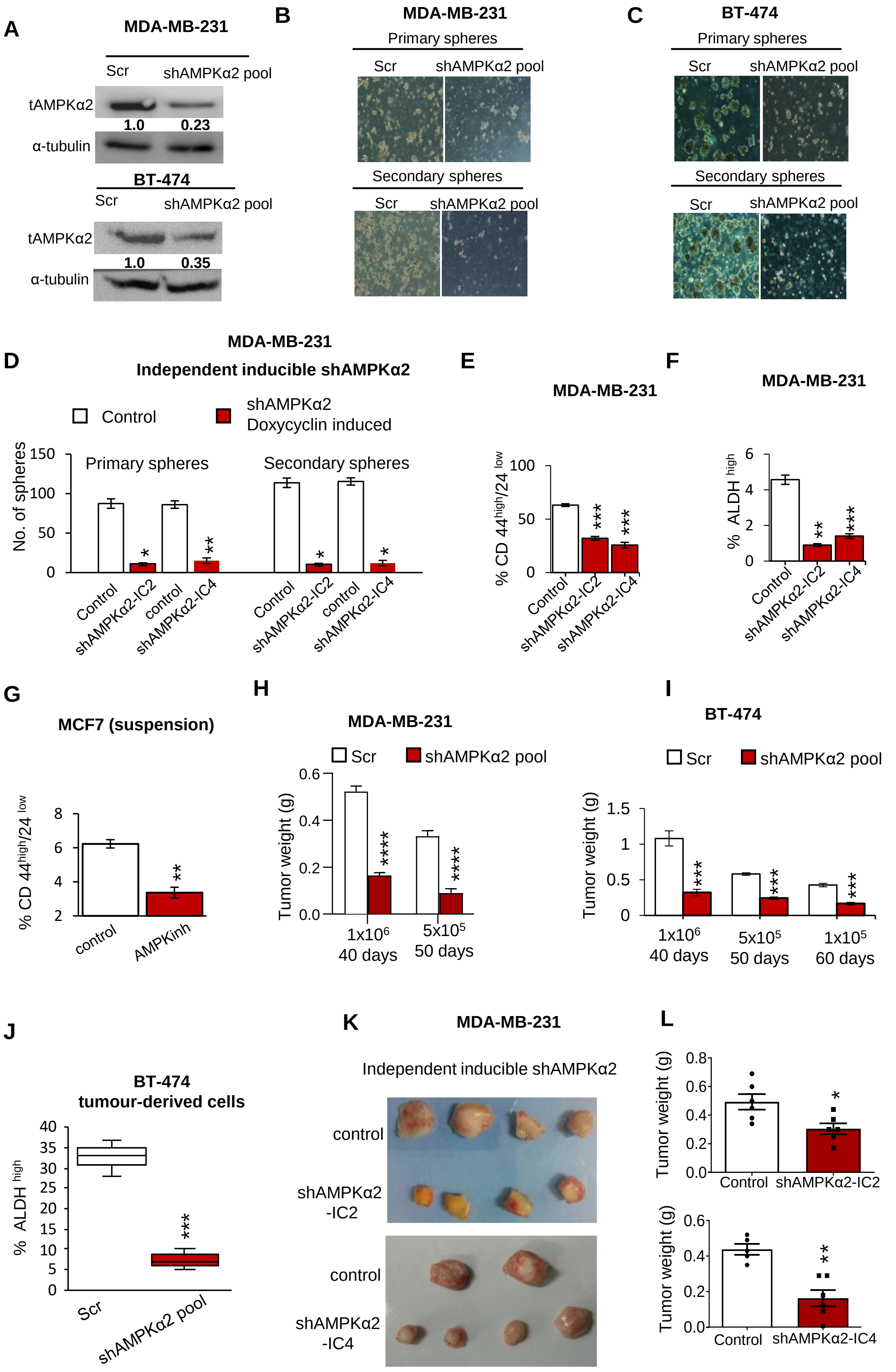

Disease Models & Mechanisms • Supplementary information

Fig. S1. A) MDA-MB-231 and BT-474 cells stably expressing shRNA against AMPKα2-pool or scrambled shRNA (Scr) were cultured in suspension for 72 hours, were harvested and subjected to western blot analysis for specified antibodies. n=3.
B and C) Representative phase contrast images (10X magnification) of MDA-MB-231 cells (B) and BT-474 cells (C) stably expressing shRNA against AMPKα2-pool or scrambled shRNA (Scr) seeded in methyl cellulose for primary and secondary sphere formation (group of cells more than 30 cells are counted as one sphere). Then spheres were counted and images were captured after a specified time period.
D, E and F) MDA-MB-231 cells stably expressing (inducible) shRNA against AMPKα2 (IC2 or IC4) or control cells were treated with doxycycline and subjected to methyl cellulose assay for primary and secondary sphere formation and no. of spheres were measured after a week (D). MDA-MB-231 cells stably expressing (inducible) shRNA against AMPKα2 (IC2 or IC4) or control cells were treated with doxycycline and cultured in suspension for 72 hours, were harvested and subjected to $CD44^{high}/24^{low}$ analysis (**E**) and ALDH assay (**F**) analysis. Error bar represents SEM; n=3.
G) MCF-7 cells were cultured in suspension in the presence of AMPK inhibitor (Compound C) for 72 hours, were harvested and subjected to $CD44^{high}/24^{low}$ analysis. DMSO served as a vehicle control. Error bar represents SEM; n=3
H-J) MDA-MB-231 ($1x10^6$ and/or $5x10^5$) cells (H) and BT-474 ($1x10^6$, or, $5x10^5$ and/or $1x10^5$) cells (I) stably expressing shRNA against AMPKα2-pool or scrambled shRNA (Scr) were injected subcutaneously into 5 female nude mice (Scr cells in the left flank, and shAMPKα2 cells in the right flank-for each cell type and each dilution) and tumour formation was monitored for a specified time period, further, tumours were isolated and weighed on specified days for tumour weight analysis. Cells were derived from the isolated tumours and subjected to ALDH assay analysis (J).
K) MDA-MB-231 cells stably expressing (inducible) shRNA against AMPKα2 (IC2 or IC4) or control cells were injected ($5x10^5$) subcutaneously into 5 female nude mice (Control cells in the left flank, and shAMPKα2 cells in the right flank). After tumors reaching $100mm^3$, mice were treated with doxycycline for induction and tumour formation was monitored for 30 days, further, tumours were isolated. Representative tumor images were captured (K) and tumor weights were plotted (L) for statistical significance.
Statistical test: Two-way ANOVA or unpaired-t test was performed for statistical significance

Disease Models & Mechanisms • Supplementary information

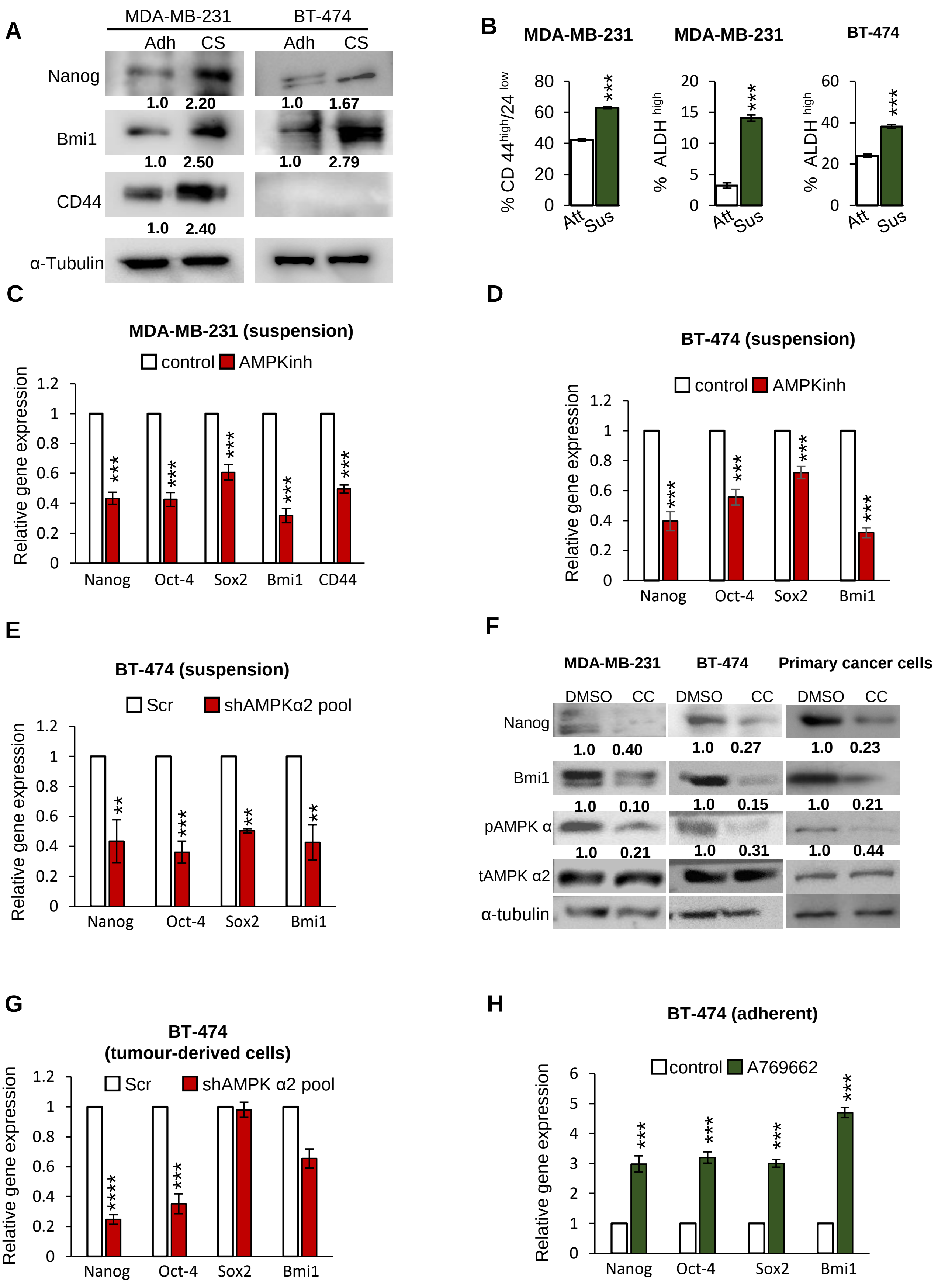

Disease Models & Mechanisms • Supplementary information

Fig. S2. A and B) MDA-MB-231 and BT-474 were seeded in adherent and suspension, after 7 days, cells were harvested and subjected to western blot analysis (A). MDA-MB-231 and BT-474 cells were seeded in adherent and suspension condition for 72 hrs, thereafter cells were subjected to FACS analysis (B).

C) MDA-MB-231 cells were cultured in suspension for 72 hours in the presence of AMPK inhibitor (Compound C), then cells were harvested and subjected to qRT-PCR analysis for specified primer sets. DMSO served as vehicle control. Error bar represents SEM; n=3.

D) BT-474 cells cultured in suspension for 72 hours in the presence of AMPK inhibitor (Compound C) were harvested and subjected to qRT-PCR analysis for specified primer sets. DMSO served as vehicle control. Error bar represents SEM; n=3.

E) BT-474 cells stably expressing shRNA against AMPKα2-pool or scrambled shRNA (Scr) were cultured in adherent condition or suspension for 72 hours, and then cells were harvested and subjected to qRT-PCR analysis for specified primer sets. Error bar represents SEM; n=3.

F) MDA-MB-231, BT-474 and primary breast cancer cells were cultured in suspension for 72 hours in the presence of AMPK inhibitor (Compound C) and then cells were harvested and subjected to western blot analysis for specified antibodies. n=3.

G) BT-474 cells stably expressing shRNA against AMPKα2-pool or scrambled shRNA cells (Scr) were injected ($1x10^6$) subcutaneously into 5 female nude mice and tumour formation was monitored for a period of 40 days. After specified time, tumours were isolated and subjected to qRT-PCR analysis for specified primer sets.

H) Adherent BT-474 cells were cultured in for 72 hours in the presence of AMPK activator (A76) and then cells were harvested and subjected to qRT-PCR analysis for specified primer sets. DMSO served as vehicle control. Error bar represents SEM; n=3.

Statistical test: Two-way ANOVA or unpaired-t test was performed for statistical significance

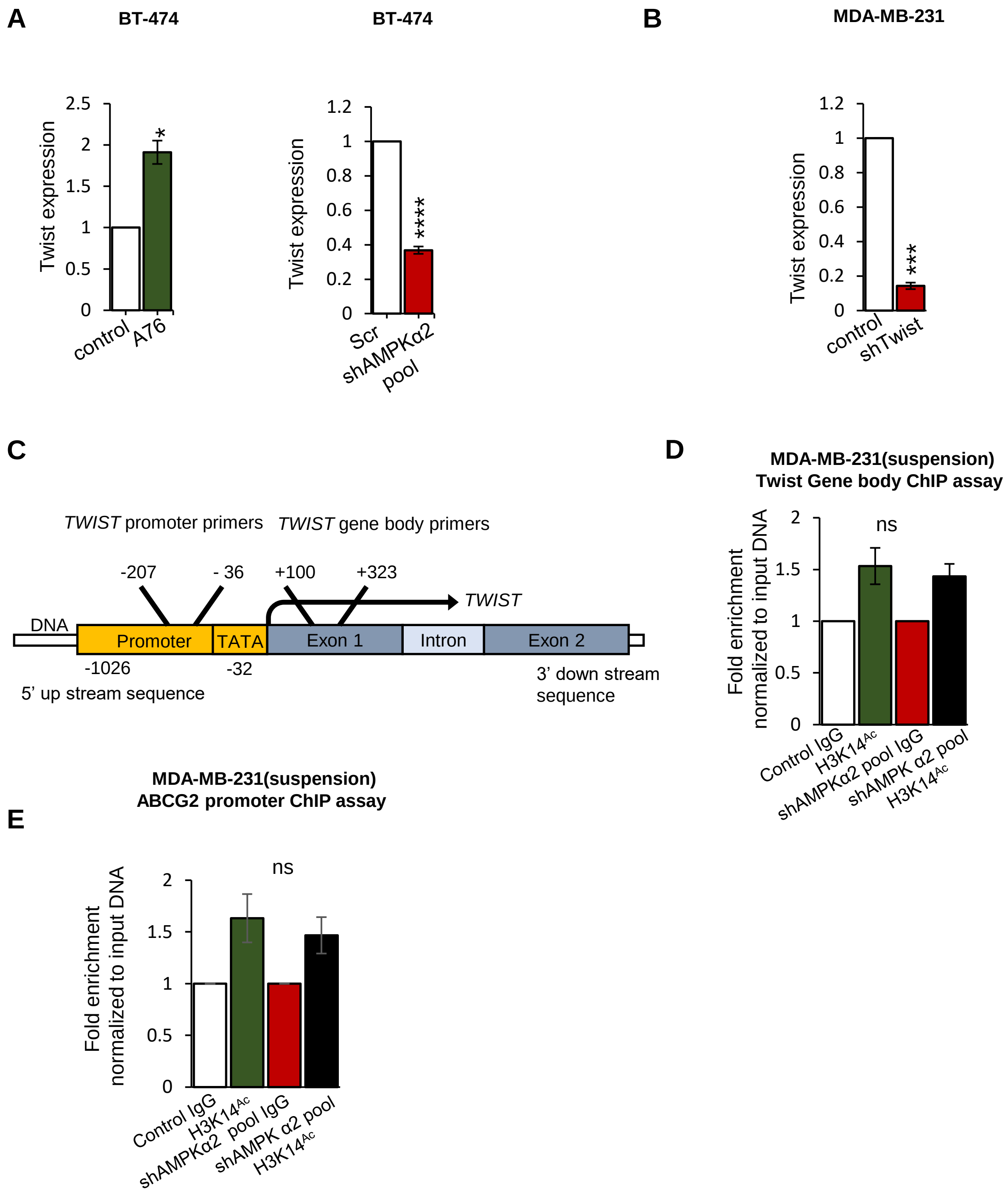

Disease Models & Mechanisms • Supplementary information

Fig. S3. A) Adherent BT-474 cells were treated with AMPK activator for 72 hours and suspension BT-474 cells stably expressing short hairpin RNA against AMPKα2-pool or scrambled shRNA (Scr) were cultured for 72 hours then harvested and subjected to qRT-PCR for Twist. Error bar represents SEM; n=4.
B) MDA-MB-231 cells (suspension) stably expressing short hairpin RNA against Twist or GFP (control) were cultured for 72 hours then harvested and subjected to qRT-PCR for Twist. Error bar represents SEM; n=4.
C) Schematic structure of the 5' upstream regulatory region of Twist and the positions of the promoter, TATA box, exons, intron and designed primer sequence locations. In the ChIP assay Twist promoter primer and gene body primer sets were used to amplify the promoter and gene body region.
D and **E)** MDA-MB-231 cells stably expressing shRNA against AMPKα2-pool or scrambled shRNA (Scr) were cultured in suspension for 72 hours, were harvested and subjected to ChIP assay using H3K14ac antibody. IgG or beads alone served as negative controls for pull-down, input DNA served as a positive control for qPCR. qRT-PCR analysis was carried out for indicated primer sets.
Statistical test: Two way ANOVA or unpaired-t test was performed for statistical significance

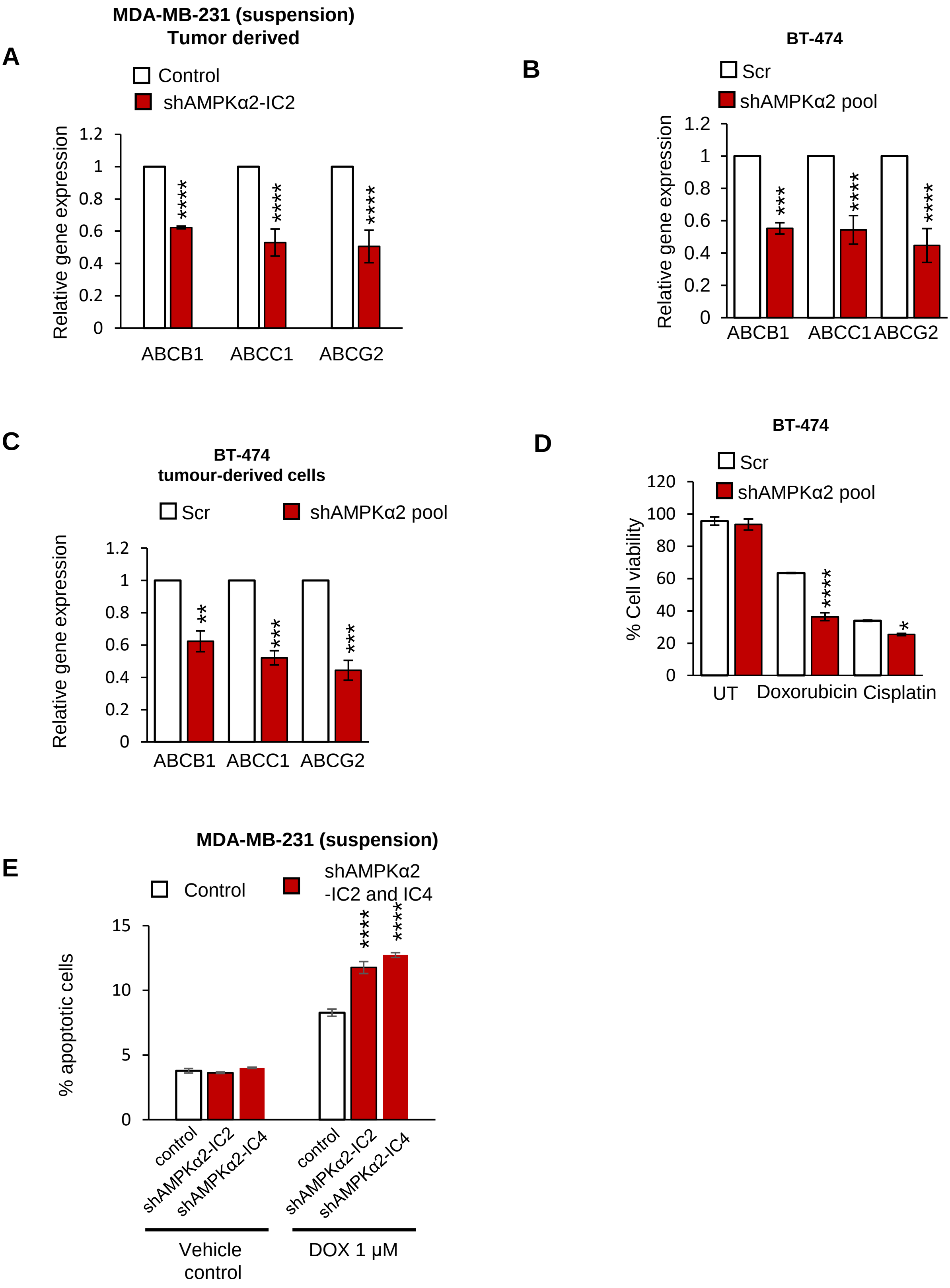

Disease Models & Mechanisms • Supplementary information

Fig. S4. A) MDA-MB-231 cells stably expressing shRNA against AMPKα2-IC2 or control cells were injected (5x105) subcutaneously into 5 female nude mice (control cells in the left flank, and shAMPKα2-IC2 cells were in the right flank). After tumors reaching 100mm3 mice were treated with doxycycline for induction and tumour formation was monitored for a period of 30 days. After specified time of tumour formation (30 days), tumours were isolated and subjected for qRT-PCR analysis for specified primer sets. Error bar represents SEM; n=3

B) BT-474 cells stably expressing shRNA against AMPKα2-pool or scrambled (Scr) were seeded in suspension, cells were harvested at the end of 72 hours for qRT-PCR analysis for specified primer sets. Error bar represents SEM; n=3.

C) BT-474 cells stably expressing shRNA against AMPKα2-pool or scrambled (Scr) were injected (1x106) subcutaneously into 5 female nude mice and tumour formation was monitored for a period of 40 days. After specified time, tumours were isolated and tumours were subjected for qRT-PCR analysis for specified primer sets. Error bar represents SEM; n=3.

D) Adherent BT-474 cells stably expressing short hairpin RNA against AMPKα2-pool or scrambled shRNA (Scr) were treated with various concentrations of doxorubicin (Dox) or cisplatin for 48 hours, and were subjected to cell viability assay (MTT). Error bar represents SEM; n=4.

E) MDA-MB-231 cells stably expressing (inducible) shRNA against AMPKα2 (IC2 or IC4) or control cells were treated with doxycycline and cultured in suspension for 72 hours, were harvested and subjected to annexin V assay. Error bar represents SEM; n=3.

Statistical test: Two-way ANOVA was performed for statistical significance

Disease Models & Mechanisms • Supplementary information

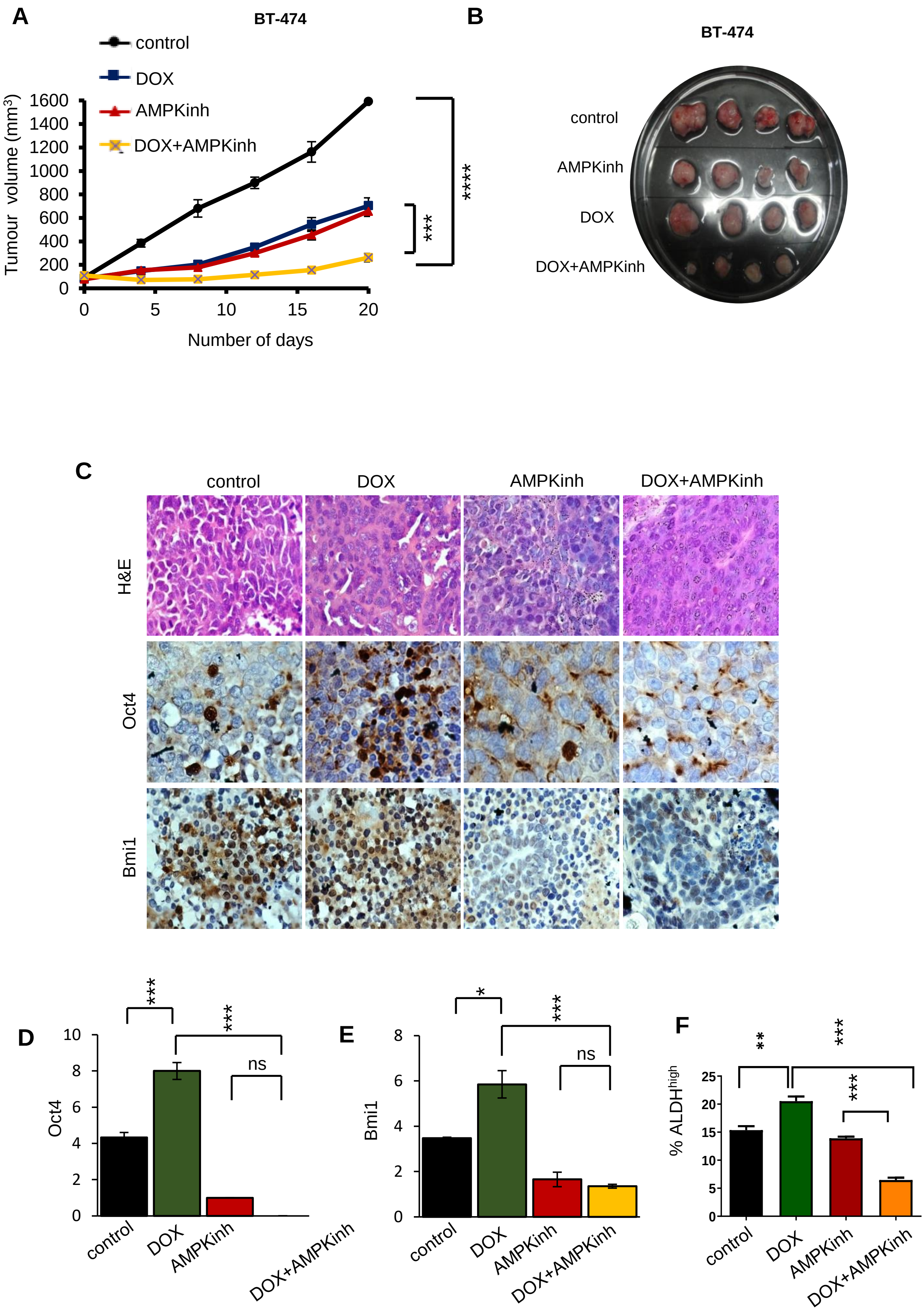
A
BT-474
control
DOX
AMPKinh
DOX+AMPKinh
Tumour volume (mm3)
Number of days
****
***
B
BT-474
control
AMPKinh
DOX
DOX+AMPKinh
C
control
DOX
AMPKinh
DOX+AMPKinh
H&E
Oct4
Bmi1
D
Oct4
***
***
ns
control
DOX
AMPKinh
DOX+AMPKinh
E
Bmi1
*
***
ns
control
DOX
AMPKinh
DOX+AMPKinh
F
% ALDHhigh
**
***
***
control
DOX
AMPKinh
DOX+AMPKinh

Fig. S5. A and B) BT-474 cells were injected into 16 female nude mice, after primary tumour formation (approximately 100 mm^3 of tumour) mice were randomized into 4 groups (4 mice/group) and treated with AMPK inhibitor or doxorubicin (Dox) or both (AMPK inhibitor and Dox) for 3 weeks. DMSO served as a vehicle control. Tumour kinetics was measured (A) for a period of 21 days and tumours were isolated and imaged (B).

C, D and E) BT-474 cells were injected into 16 female nude mice, after primary tumour formation (approximately 100 mm^3) mice were randomized into 4 groups (4 mice/group) and treated with AMPK inhibitor or doxorubicin (Dox) or both (AMPK inhibitor and DOX) for 3 weeks. DMSO served as vehicle control. 21 days, mouse tumours were resected and subjected to H&E staining and IHC analysis for the expression of Oct4 and Bmi1. Oct4 (D) and Bmi1 (E) expression was semi-quantitatively assessed by senior pathologist in a blinded fashion. Another part of tumor tissues were processed and single cells were isolated and subjected to ALDH analysis (F). Error bar represents SEM; n=6.

Statistical test: Two-way ANOVA was performed for statistical significance

Disease Models & Mechanisms • Supplementary information

Table S1. Cancer tissue sample details, ER: Estrogen receptor, PR: Progesterone receptor, HER2: Human epidermal growth factor receptor 2, TNBC, Triple negative breast cancer.

S. no	Tissue code	Breast cancer sub-type	Chemotherapy status	Age of the patient	lymph node status
1	BCT01	ER/PR Positive	Chemo-naive	48	Positive
2	BCT02	Her2 Positive	Chemo-naive	49	Positive
3	BCT03	Her2 Positive	Chemo-naive	40	Negative
4	BCT04	ER/PR Positive	Chemo-treated	51	Positive
5	BCT05	TNBC	Chemo-treated	38	Positive
6	BCT06	TNBC	Chemo-treated	52	Positive
7	BC07	ER/PR Positive	Chemo-treated	38	Positive
8	BCT08	Her2 Positive	Chemo-naive	57	Negative
9	BCT09	TNBC	Chemo-naive	45	Positive
10	BCT10	TNBC	Chemo-treated	51	Positive
11	BCT11	ER/PR Positive	Chemo-treated	36	Positive
12	BCT12	Her2 Positive	Chemo-naive	53	Negative
13	BCT13	TNBC	Chemo-naive	71	Positive
14	BCT14	TNBC	Chemo-treated	65	Positive
15	BCT15	ER/PR Positive	Chemo-treated	68	Negative
16	BCT16	TNBC	Chemo-naive	35	Negative
17	BCT17	Her2 Positive	Chemo-treated	53	Positive
18	BCT18	ER/PR Positive	Chemo-treated	57	Positive
19	BCT19	TNBC	Chemo-naive	51	Positive
20	BCT20	Her2 Positive	Chemo-naive	85	Positive
21	BCT21	ER/PR Positive	Chemo-naive	39	Negative
22	BCT22	TNBC	Chemo-treated	48	Positive
23	BCT23	Her2 Positive	Chemo-treated	42	Negative
24	BCT24	Her2 Positive	Chemo-naive	45	Positive
25	BCT25	ER/PR Positive	Chemo-treated	50	Negative
26	BCT26	ER/PR Positive	Chemo-naive	59	Positive
27	BCT27	TNBC	Chemo-naive	62	Positive
28	BCT28	TNBC	Chemo-treated	45	Positive
29	BCT29	ER/PR Positive	Chemo-treated	61	Negative
30	BCT30	ER/PR Positive	Chemo-naive	35	Negative
31	BCT31	Her2 Positive	Chemo-naive	48	Positive
32	BCT32	ER/PR Positive	Chemo-naive	46	Positive
33	BCT33	TNBC	Chemo-treated	41	Positive
34	BCT34	ER/PR Positive	Chemo-treated	39	Positive
35	BCT35	ER/PR Positive	Chemo-treated	54	Positive
36	BCT36	TNBC	Chemo-naive	65	Positive
37	BCT37	Her2 Positive	Chemo-naive	57	Positive
38	BCT38	TNBC	Chemo-treated	55	Positive
39	BCT39	Her2 Positive	Chemo-treated	52	Negative
40	BCT40	TNBC	Chemo-treated	39	Positive
41	BCT41	TNBC	Chemo-naive	46	Positive
42	BCT42	TNBC	Chemo-naive	42	Positive

Disease Models & Mechanisms • Supplementary information

Supplementary Materials and Methods

A Aldehyde dehydrogenase activity assay

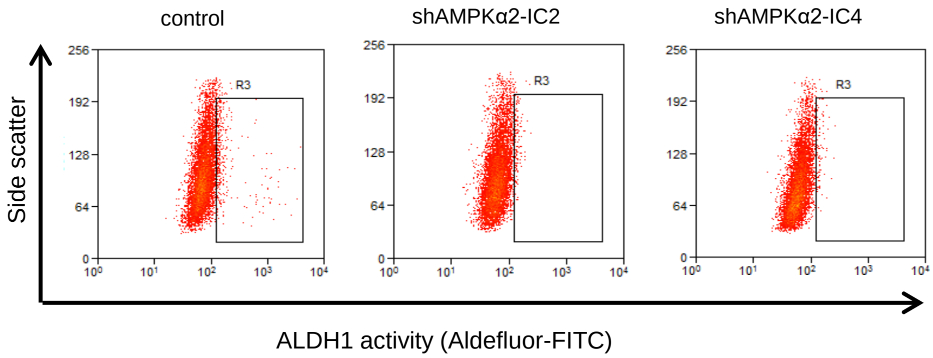

A) MDA-MB-231 cells stably expressing 2 independent sequences of shRNA against AMPKα2 (IC-2 or IC-4) in an doxycycline inducible backbone (pTRIPZ) or control non-targeting shRNA were induced with doxycycline and cultured in suspension for 72 hours. Cells were harvested and subjected to ALDH1 activity assay using Aldefluour kit (Stemcell technologies) as per manufacturer's instructions. Representative FACS dot plots are shown.

B $CD44^{high}/24^{low}$ assay

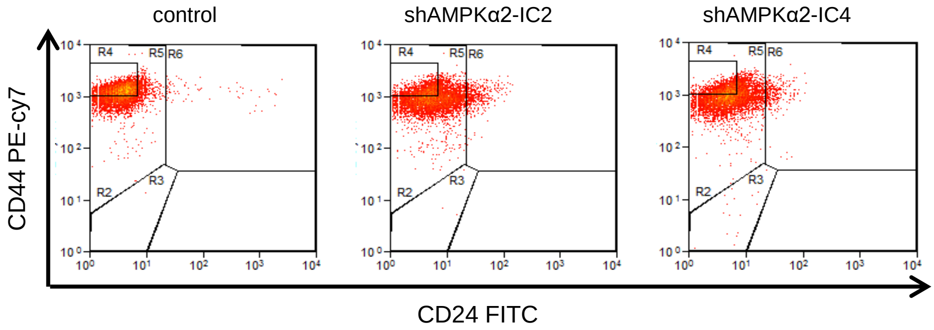

B) MDA-MB-231 cells stably expressing 2 independent sequences of shRNA against AMPKα2 (IC-2 or IC-4) in an doxycycline inducible backbone (pTRIPZ) or control non-targeting shRNA were induced with doxycycline and cultured in suspension for 72 hours. Cells were harvested and stained with CD44 and CD24 antibodies conjugated to fluorophores PE-cy7 and FITC respectively. Representative FACS dot plots are shown.

Disease Models & Mechanisms • Supplementary information
